# Disulphide bond-forming enzymes in clostridial species

**DOI:** 10.1099/mic.0.001603

**Published:** 2025-09-12

**Authors:** Claudia Antonika, Jocelyne Mendoza, Cristina Landeta

**Affiliations:** 1 Department of Biology, Indiana University, Bloomington, USA

**Keywords:** *Clostridioides difficile*, *Clostridium botulinum*, *Clostridium tetani*, cystine, disulphide bond, DsbA, DsbB, ergothioneine, glutathione, low molecular weight thiol, VKOR

## Abstract

Disulphide bond formation is critical for the folding and stability of proteins involved in bacterial cell envelope processes yet remains understudied in clostridial pathogens. While a few Clostridia-derived toxins and virulence factors are known to depend on disulphide bonds, the enzymes catalysing their formation are poorly characterized. Here, we performed a bioinformatic search to identify ten putative disulphide bond-forming enzymes in Clostridia. We cloned and codon-optimized these genes, testing their ability to complement *Escherichia coli dsb* mutants. Our analysis revealed a VKOR homologue, a VKOR-DsbA fusion and three DsbA homologues capable of complementing *E. coli dsb* mutants. Notably, *Clostridium botulinum* DsbA functioned independently of a regenerating partner, with its activity recycled by glutathione disulphide or ergothioneine. In contrast, *Clostridium tetani* and *Clostridioides difficile* DsbA proteins required *E. coli* DsbB for regeneration, suggesting reliance on distinct thiol or enzyme partners. Understanding oxidative protein folding in Clostridia could reveal new targets for antibacterial intervention.

## Importance

Oxidative protein folding is crucial for bacterial survival and virulence. Several pathogenic Clostridia species – such as the agents of tetanus, botulism and *Clostridioides difficile* colitis – produce disulphide-bonded virulence factors, including tetanus and botulinum neurotoxins. However, the enzymatic systems responsible for their folding remain largely uncharacterized. In this study, we identified putative disulphide bond catalysts in Clostridia, revealing potential differences in their regeneration mechanisms and suggesting reliance on distinct thiol or enzyme partners. Understanding oxidative protein folding in Clostridia could reveal novel targets for antibacterial development.

## Introduction

Disulphide bond formation has a central role in protein folding of both eukaryotes and prokaryotes. In bacteria, disulphide bonds play a role in the folding and stability of proteins involved in important cellular processes such as cell division and assembly of the outer membrane of Gram-negative bacteria. In addition, many virulence proteins that are exported from the cytoplasm to either the bacterial cell envelope or into host cells during pathogenesis require the stability conferred by disulphide bonds in destabilizing environments. In fact, studies with animal models have shown that mutations in DsbA and DsbB decrease the virulence in several pathogenic bacteria (reviewed by Heras *et al*. and Landeta *et al*. [1,2]). Recent studies have also shown that inhibiting disulphide bond formation can re-sensitize multidrug-resistant clinical isolates of *Escherichia coli*, *Klebsiella pneumoniae* and *Stenotrophomonas maltophilia* to currently available antibiotics [3,4].

**Fig. 1.**
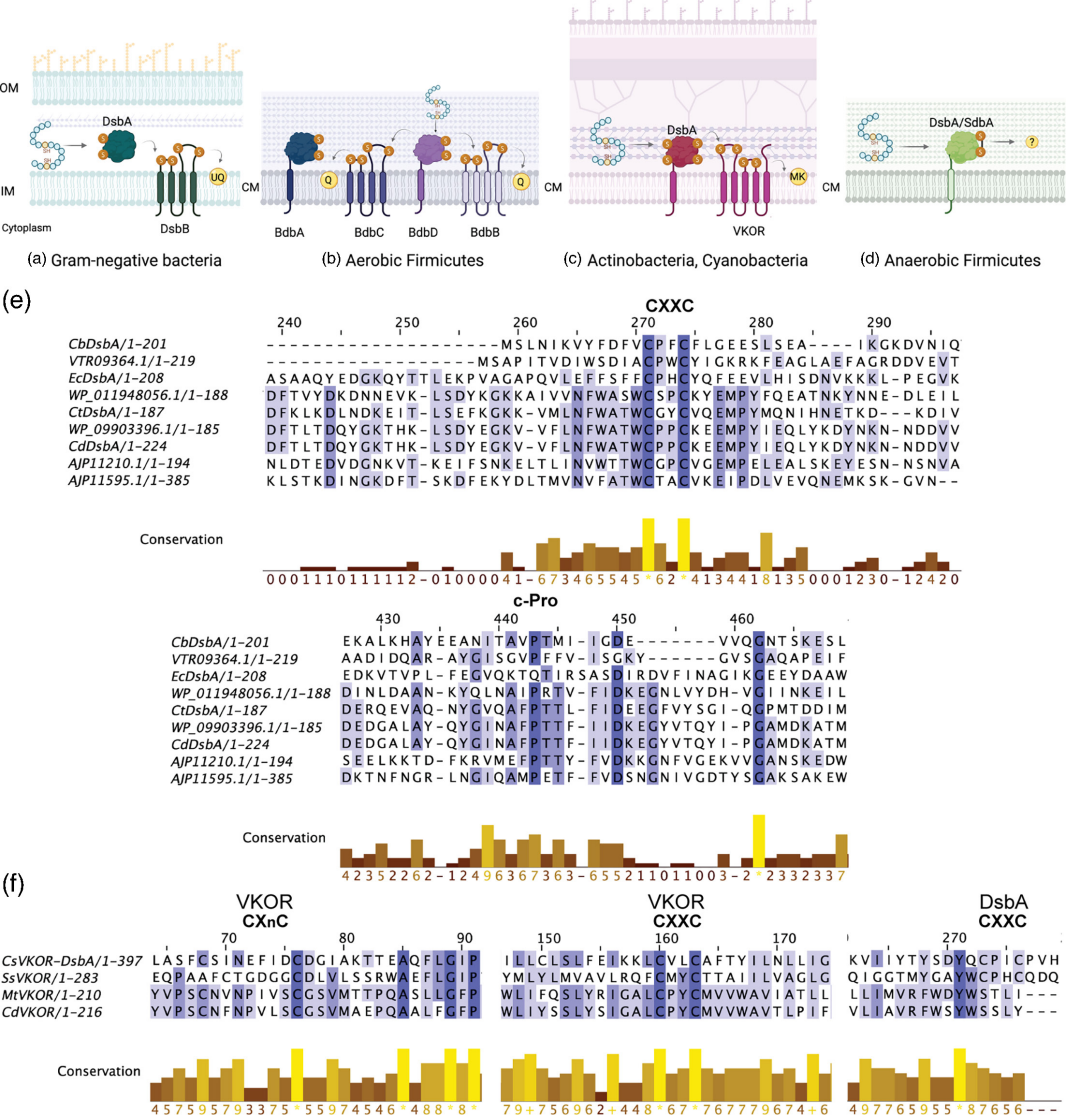
Disulphide bond formation takes place in the bacterial cell envelope. Catalysts involved in oxidative protein folding are shown for model organisms: *E. coli*, Gram-negative bacteria (**a**)*; Bacillus subtilis*, aerobic Firmicutes (**b**)*; Mycobacterium tuberculosis,* actinobacteria, cyanobacteria (**c**); and *Staphylococcus aureus,* anaerobic Firmicutes (**d**). See text for further details. Grey arrows indicate electron flow. UQ, ubiquinone; Q, quinone; MK, menaquinone; and (?), yet to-be-identified oxidant. Created in BioRender. (e) Portions of multiple sequence alignments with potential DsbA enzymes from Clostridia. The protein name or identifier used refers to the findings of this work. The alignment displays the Cys-XX-Cys motif (CXXC) and cis-Proline (c-Pro) residue conservation. *Cb*, *Clostridium botulinum*; *Ec*, *E. coli*; *Ct*, *Clostridium tetani*; and *Cd*, *Clostridioides difficile*. (f) Portions of the multiple sequence alignments with potential VKOR enzymes from Clostridia. The protein name or identifier used refers to the findings of this work. The alignment displays the CXXC and CXnC conservation. The protein fusions VKOR-DsbA show the additional CXXC motif conservation. *Cs*, *Clostridium* sp.; *Ss*, *Synechococcus* sp.; *Mt*, *Mycobacterium tuberculosis*; and *Cd*, *Clostridioides difficile*. Alignments were done with Clustal Omega [67] using Jalview [68] as a display.

The formation of disulphide bonds in proteins is an oxidative process that generates a covalent bond linking the sulphur atoms of two cysteine residues. Disulphide-bonded proteins are located in more oxidizing environments such as the bacterial cell envelope. There are two main systems known to introduce disulphide bonds in bacteria. The two systems share the first substrate oxidation step, but each system differs in the second step which recycles the first enzyme’s activity (Fig. 1). The first step in *E. coli* starts with DsbA, a periplasmic thioredoxin homologue, which introduces disulphide bonds into substrates through its redox-active Cys-XX-Cys (CXXC) motif, forming an unstable mixed-disulphide complex [5]. The resolution of this complex by a disulphide exchange reaction leaves the disulphide-bonded substrate and reduced DsbA [6]. Subsequently, the membrane protein DsbB recycles DsbA’s activity by transferring the two gained electrons to quinones by *de novo* disulphide bond formation through its CXXC motif [7,11] (Fig. 1a). While most Proteobacteria and aerobic Gram-positive Firmicutes are predicted to use DsbA- and DsbB-like proteins to form disulphide bonds in exported proteins (Fig. 1b), Actinobacteria, Cyanobacteria, aerobic δ-proteobacteria and Spirochaetes in the genus *Leptospira* maintain DsbA oxidized not by DsbB, but by the protein VKOR, a homologue of human vitamin K epoxide reductase [12] (Fig. 1c). Similar to DsbB, VKOR regenerates DsbA’s activity using quinone cofactors [13].

Generally, bacteria with significantly more even numbers of cysteines in exported proteins also possess DsbA and DsbB/VKOR homologues, suggesting that oxidative protein folding occurs in these organisms. However, the extent of oxidative protein folding varies among species. For instance, *E. coli* is predicted to introduce disulphide bonds into ~13% of its proteome [12], while mycobacteria oxidize ~14% of its proteome [14]. In contrast, obligate anaerobic organisms tend to exclude cysteines in exported proteins [12,15]. Despite this, some of these organisms secrete fewer disulphide-bonded proteins, which are folded by DsbA-like proteins but lack a regenerating enzyme partner [16]. A notable example is *Streptococcus gordonii* DsbA-like protein, SdbA, which belongs to the AhpC/TSA family and can catalyse oxidation with one single cysteine of its CXXC motif (Fig. 1d) [17,18]. Similarly, the facultative aerobic Gram-positive pathogen *Staphylococcus aureus* has been reported to rely solely on a DsbA protein that is possibly regenerated by small molecule oxidants such as molecular oxygen [19].

The Clostridia includes organisms that are strict fermentative anaerobes that typically produce hydrogen, organic solvents and/or organic acids as fermentation by-products. The class includes several human pathogens such as the agents of tetanus and botulism as well as *Clostridioides difficile* colitis. Cysteine and other thiols play a central role in *C. difficile* metabolism. *C. difficile* indeed requires large amounts of cysteine for optimal growth [20], with its production of toxins dependent on cysteine availability [21,22]. Furthermore, some *C. difficile* strains show an astonishing tolerance to oxygen for virtually strict anaerobic bacteria, which may be linked to their thiol metabolism [23]. A cysteine labelling study in *C. difficile* proteome found that at least 10% of peptides are highly oxidized [24]. In addition, there are a few known clostridial-derived disulphide-bonded proteins with pathogenic-related functions, including the tetanus and botulinum neurotoxins [25,26], TcdAB toxins [27] and a cysteine-rich protein (CD1067, CdeC) present in *C. difficile* spores [28,29]. We hypothesized that Dsb catalysts may play a role in the formation of disulphide bonds in Clostridia and presumably low molecular weight (LMW) thiols are involved in the electron transfer, given the lack of quinones in these organisms [30]. The LMW thiols could be synthesized by the organism such as glutathione disulphide (oxidized glutathione) and cystine (oxidized cysteine), or through acquisition from their environment, such as ergothioneine (oxidized ergothioneine), which has been recently shown to be imported to the cytoplasm in Clostridia and other gastrointestinal microbes [31,32].

The study of diverse Dsb catalysts in *E. coli* is possible despite the lack of homology between VKOR and DsbB proteins; both DsbB and VKOR homologues from Gram-negative bacteria, actinobacteria such as *Mycobacterium tuberculosis*, archaea and two eukaryotes can complement disulphide bond formation in *E. coli* [12,33,36]. Similarly, DsbA proteins have been shown to substitute *E. coli* DsbA’s function [37,40]. Thus, disulphide exchange reactions are widely conserved and can be effectively studied in *E. coli*.

In this work, we sought to find disulphide bond catalysts in clostridial species and assess their ability to complement the disulphide bond formation of *E. coli dsb* mutants. We identified a VKOR homologue, a VKOR fused to DsbA and three DsbA homologues in clostridial genomes that can complement *E. coli dsb* mutants. Additionally, we demonstrated that *Clostridium botulinum* DsbA can function with or without a regenerating partner, and its activity can be recycled by LMW thiols. Conversely, *Clostridium tetani* and *C. difficile* DsbA proteins were regenerated only by *E. coli* DsbB.

## Methods

### Strains and growth conditions

The strains and plasmids used in this study are listed in Table 1. The synthetic gene fragments and primers used in this study are listed in Tables2  3, respectively. To construct the plasmids used in this work, the vectors (pTrc99a or pDSW204) were amplified with primers PR24 and PR25 using Q5 high-fidelity polymerase (New England Biolabs). The DNA fragments were designed for *E. coli* using Integrated DNA Technologies (IDT) Codon Optimization Tool and synthesized by IDT (gblocks, Table 2), and the signal sequences or transmembrane (TM) segments were removed and fused to *E. coli* DsbA signal peptide (19 aa). *E. coli* DsbA was amplified with primers PR3 and PR8 from *E. coli* HK295 genome, while *M. tuberculosis* DsbA was amplified without its TM segment using primers PR11 and PR12 from *M. tuberculosis* H37Rv genome. To amplify *M. tuberculosis* VKOR, primers PR94 and PR95 were used using *M. tuberculosis* H37Rv genome. The *M. tuberculosis* VKOR was then ligated to a PCR product containing the TetR encoding gene and the Tet promoter (P_Tet_) from pAJM717 [41] using primers PR92 and PR93. The TetR-P_tet_-MtVKOR block was then ligated to the PCR vector amplified with primers PR90 and PR91 using PL16 as template. A terminator was added to the resulting vector using primers PR112 and PR113 using KLD enzyme mix (New England Biolabs). The gblocks or PCR products were ligated using NEBuilder® HiFi Assembly (New England Biolabs). To clone *Cd*VKOR into PL188, PL189, PL190, PL192 and PL194, *Cd*VKOR was amplified using primers PR685 and PR686 using PL42 as template. The insert and plasmids were then digested with BamHI and XbaI and ligated with T4 Ligase (New England Biolabs). Plasmids were verified by PCR using primers PR20 and PR21 and by Sanger sequencing with PR21. All plasmids were transformed to HK317 (Δ*dsbA*), HK320 (Δ*dsbB*) or HK329 (Δ*dsbA*Δ*dsbB*).

**Table 1.** List of strains and plasmids used in this study

ID	Genotype	Ref.
*Strains*		
HK295	*E. coli* MC1000 Δ*ara*714 *leu*^+^	Beckwith lab
HK314	HK295 λMalFlacZ102	[69]
HK317	HK295 Δ*dsbA*	[69]
HK320	HK295 Δ*dsbB*	[69]
HK329	HK295 ΔdsbAΔ*dsbB*	[69]
HK361	HK295 Δ*dsbA*, λMalFlacZ102	[69]
CL499	HK295 λMalFlacZ102, pTrc99a	[33]
LL40	HK295 Δ*dsbA*, λMalFlacZ102, pTrc99a	This study
CL379	HK295 Δ*dsbB*, λMalFlacZ102, pTrc99a	[33]
High expression		
LL27	HK295 Δ*dsbB*, pTrc99a-VTR07230.1 (*Cd*VKOR)	This study
LL26	HK295 Δ*dsbA*Δ*dsbB*, pTrc99a-R7I132_9CLOT (*Cs*VKOR-DsbA)	This study
LL22	HK295 Δ*dsbA* pTrc99a-WP_011948056.1	This study
LL23	HK295 Δ*dsbA*, pTrc99a-WP_011948075.1 (*Cb*DsbA)	This study
LL59	HK295 Δ*dsbA*Δ*dsbB*, pTrc99a-WP_011948075.1 (*Cb*DsbA)	This study
LL24	HK295 Δ*dsbA*, pTrc99a-AJP11210.1	This study
LL28	HK295 Δ*dsbA*, pTrc99a- AJP11595.1	This study
LL25	HK295 Δ*dsbA*, pTrc99a-WP_09903396.1	This study
LL109	HK295 Δ*dsbA*, pTrc99a- EQE38533.1 (*Cd*DsbA)	This study
LL607	HK295 Δ*dsbA*Δ*dsbB*, pTrc99a-EQE38533.1 (*Cd*DsbA)	This study
LL272	HK295 Δ*dsbA*, pTrc99a-VTR09364.1	This study
LL609	HK295 Δ*dsbA*Δ*dsbB*, pTrc99a-VTR09364.1	This study
LL29	HK295 Δ*dsbA*, pTrc99a-AAO34798.1 (*Ct*DsbA)	This study
LL606	HK295 Δ*dsbA*Δ*dsbB*, pTrc99a-AAO34798.1 (*Ct*DsbA)	This study
LL687	HK295 Δ*dsbA*Δ*dsbB*, pTrc99a-*MtdsbA*	This study
CL382	HK295 Δ*dsbB*, λMalFlacZ102, pTrc99a-*Mtvkor*	[33]
Lower expression		
LL267	HK295 Δ*dsbA*, pDSW204-WP_011948056.1	This study
LL268	HK295 Δ*dsbA*, pDSW204-AJP11210.1	This study
LL271	HK295 Δ*dsbA*, pDSW204-AJP11595.1	This study
LL269	HK295 Δ*dsbA*, pDSW204-WP_09903396.1	This study
LL270	HK295 Δ*dsbA*, pDSW204-EQE38533.1	This study
LL608	HK295 Δ*dsbA*Δ*dsbB*, pDSW204-EQE38533.1	This study
LL273	HK295 Δ*dsbA*, pDSW204-AAO34798.1 (*Ct*DsbA)	This study
LL16	HK295 Δ*dsbA*, pDSW204-*EcdsbA*	This study
LL686	HK295 Δ*dsbA*Δ*dsbB*, pDSW204-*EcdsbA*	This study
LL103	HK295 Δ*dsbA*Δ*dsbB*, pDSW204-*MtdsbA*-TetR-P_tet_-*Mtvkor*	This study
LL18	HK295 Δ*dsbA*, pDSW204- *MtdsbA*	This study
*Plasmids*		
pTrc99a	Expression vector, Amp^r^	[70]
pDSW204	Promoter down mutation in −35 of pTrc99a, Amp^r^	[71]
pAJM.717	P_tet_*-YFP; inducible with aTc	[41]
PL42	pTrc99a-VTR07230.1 (*Cd*VKOR)	This study
PL39	pTrc99a-R7I132_9CLOT (*Cs*VKOR-DsbA)	This study
PL35	pTrc99a-WP_011948056.1	This study
PL36	pTrc99a-WP_011948075.1 (*Cb*DsbA)	This study
PL37	pTrc99a-AJP11210.1	This study
PL43	pTrc99a-AJP11595.1	This study
PL38	pTrc99a-WP_09903396.1	This study
PL83	pTrc99a-EQE38533.1 (*Cd*DsbA)	This study
PL193	pTrc99a-VTR09364.1	This study
PL44	pTrc99a-AAO34798.1 (*Ct*DsbA)	This study
PL1	pTrc99a-*MtdsbA*	This study
pRD33	pTrc99a-6XHis-*Mtvkor*	[72]
PL188	pDSW204-WP_011948056.1	This study
PL189	pDSW204-AJP11210.1	This study
PL192	pDSW204-AJP11595.1	This study
PL190	pDSW204-WP_09903396.1	This study
PL191	pDSW204-EQE38533.1 (*Cd*DsbA)	This study
PL194	pDSW204-VTR09364.1	This study
PL15	pDSW204-*EcdsbA*	This study
PL16	pDSW204-*MtdsbA*	This study
PL72	pDSW204-*MtdsbA*-TetR-P_tet_-6XHis-*Mtvkor*	This study
PL429	pDSW204-WP_011948056.1-*Cd*VKOR	This study
PL430	pDSW204-AJP11210.1-*Cd*VKOR	This study
PL431	pDSW204-WP_09903396.1-*Cd*VKOR	This study
PL433	pDSW204-AJP11595.1-*Cd*VKOR	This study
PL432	pDSW204-VTR09364.1-*Cd*VKOR	This study

**Table 2.** List of synthetic DNA fragments used in this study

CdVKOR (VTR07230.1, *C. difficile* str. NCTC13750)	
Gblock13	TTTCACACAGGAAACAGACC**ATG**TCTGATTCTGCCCTTACATCAAGTGAAGCTGGCCCGGATGACGACCGTGCTCATCTGGCGGAAGCGGT GCGTTCGTGGACGCGTATTGTTGCGTGGCTTTTGAGCGTCGGGGGAGCCATTGGATTTGTAGCCGCCTTCGTTCTGACTGTCGAGCGCTTC GAGTTGGCTGCGGACCCGGACTACGTCCCAAGTTGTAATTTTAACCCAGTGTTGAGCTGTGGGAGTGTAATGGCTGAACCACAAGCCGCCT TATTCGGCTTTCCTAACCCTTTGCTGGGAATCGCAGGCTTCGCCGTTGCAGTCACCACGGGTATGGCCTTACTGGCGGGGGCACGCTTAGC AGGTTGGTATTGGGCAGGATTGCAAGTTGGCGTCACTGCAGCCATGGTCTTTATCGGTTGGCTGATCTACTCTTCGTTATACTCGATTGGCGC ATTATGTCCCTATTGTATGGTCGTCTGGGCTGTCACATTACCCATTTTCGTATTCGTTTCCGTGCGTAACGCACACGCCTCAGGGTTAACATCG CGTAGCCGTCTGGCTTTGGTGTTGGCTCGCAACCACGCGCCTATCTTGGTAGTAGGAGTAGGTCTTGTTATCGTTTTAATTGCCGTGCGCTTC TGGTCCTACTGGTCTAGTTTGTAC**TGA**GAATTCGAGCTCGGTACCCG Protein sequence MSDSALTSSEAGPDDDRAHLAEAVRSWTRIVAWLLSVGGAIGFVAAFVLTVERFELAADPDYVPSCNFNPVLSCGSVMAEPQAALFGFPNPLLGIAG FAVAVTTGMALLAGARLAGWYWAGLQVGVTAAMVFIGWLIYSSLYSIGALCPYCMVVWAVTLPIFVFVSVRNAHASGLTSRSRLALVLARNHAPILVV GVGLVIVLIAVRFWSYWSSLY
CsVKOR-DsbA (R7I132_9CLOT, *Clostridium* sp. CAG:768)	
Gblock14	TTTCACACAGGAAACAGACC**ATG**CAAAAAGAAACAAAGAAAAAGGTGGCTATTGGTGTGATCAGCTTGATTGGAATTATCACCACTATCAAACT GGCAATCATTTACTATAATGCCAATTTCAATCCGTATGCGTTAGCGTCGTTTTGCTCGATCAACGAATTCATTGACTGCGACGGGATCGCTAAAA CCACCGAGGCCCAGTTCCTTGGGATTCCGTTGGCCTATTGGGGCCTGTTTTTTTACTCCTTTGTTCTGCTTATGTTGTTTGCTCAAAAGTTGAA AAATTTTAAGTTACTGAAATTCTTAGAAGTGTTTAAGAATCCGCTTGACTACATTGCAAGTCTTGGCCTTATCAGCTTCCTGATCTCAATGATTCT GTTGTGTCTTTCTCTGTTCGAGATTAAAAAATTGTGCGTTTTATGCGCGTTCACGTACATTTTAAATCTGTTGATTGGCTGCATTGCTACGAACTT TAAAGACGGCGGGTTTGTGAAGTCGATCAAACAGTCTTTCATCGACTTCTTGGATGCTGTAAAAATCAAGAAGTACTTAATTGCGTTTATCATTG TAATGCTGATTGCCGGCGGTTTCTTGGCATACACACGCATCACGTTCGTATTCGCACCACAAGTCAAACGCCAGTATGAGTTTAAGGAGTTCAC TCACAAGAAAAATAAGTACGCGGTTAAAGGTAACCTGTTAGGAGACGAAGATGCCAAAGTCATCATCTACACGTACTCTGACTATCAATGCCCCA TCTGCCCGGTACATAACAGTATGATGCATAAATTGGCCAAAGAAATGAAGGGAATTAAAATCGTTCACCGTAACCTGCCGCTGGACACAGACTGT AACAAGTATCTGCAGGGGCCGTTTCACTACGGCTCGTGCATTGATGCTCGTTACAGCATCGCCGCTGAAAAACAGGGCAAGTTATGGGACATG AACAATATGTTGTTCGAAAAAAAGCCCCAGACGGAAGAGGATGTCTTGAAGTACGCGGCCGATATGGGGTTTGATATGGAGAAGTTACAGGAGG ATGCGAACTCTCCAGAGACTCAAAAGGAGATCAAAGATCAGATTGATGATGCATATAAGCGTGGAATTCAAGGGACTCCTTCGACGATGATTAAC AATGAGGTGTATATTGGAATTAAGACCTACAAAGAGTATCAGGAGTGGGTAGAGAAGTTAGGGGCGGAAAAACGT**TGA**GAATTCGAGCTCGGTA CCCG Protein sequence MQKETKKKVAIGVISLIGIITTIKLAIIYYNANFNPYALASFCSINEFIDCDGIAKTTEAQFLGIPLAYWGLFFYSFVLLMLFAQKLKNFKLLKFLEVFKNPLDYI ASLGLISFLISMILLCLSLFEIKKLCVLCAFTYILNLLIGCIATNFKDGGFVKSIKQSFIDFLDAVKIKKYLIAFIIVMLIAGGFLAYTRITFVFAPQVKRQYEFKEF THKKNKYAVKGNLLGDEDAKVIIYTYSDYQCPICPVHNSMMHKLAKEMKGIKIVHRNLPLDTDCNKYLQGPFHYGSCIDARYSIAAEKQGKLWDMNNML FEKKPQTEEDVLKYAADMGFDMEKLQEDANSPETQKEIKDQIDDAYKRGIQGTPSTMINNEVYIGIKTYKEYQEWVEKLGAEKR
CtDsbA (AAO34798.1, *C. tetani* E88)	
Gblock12	TTTCACACAGGAAACAGACC**ATG**AAAAAGATTTGGCTGGCGCTGGCTGGTTTAGTTTTAGCGTTTAGCGCATCGGCGTTCTACGTCGATAAAAATCA GAAAGACATCGACAATAAGTCGAACACAACTACCCAAACAGAAATTAGCGAGGACAAACCGAAACGTATCGCCTCTCTGGACTTTAAACTGAAAGAC CTGAATGACAAAGAGATCACTTTGTCGGAATTCAAAGGTAAAAAGGTAATGTTGAATTTCTGGGCAACATGGTGTGGTTACTGCGTTCAGGAAATGCC TTATATGCAGAATATTCACAACGAAACGAAAGACAAAGACATTGTAATTCTTGCAGTAAACGTCGGAGAGAATAAAGACAAAATCAAAAAATTTATGGAG AAGAAATCGTACGATTTCCCGGTCGTCCTGGATGAACGCCAGGAAGTGGCCCAGAATTATGGAGTCCAAGCGTTCCCTACGACGTTATTCATCGACG AAGAAGGCTTTGTTTACAGTGGCATCCAAGGCCCAATGACGGACGACATCATGCGTAAGGAACTGAAAATTAAA**TGA**GAATTCGAGCTCGGTACCCG Protein sequence **MINLKKKVTIFLILILIILSSVFYK**FYVDKNQKDIDNKSNTTTQTEISEDKPKRIASLDFKLKDLNDKEITLSEFKGKKVMLNFWATWCGYCVQEMPYMQNIHN ETKDKDIVILAVNVGENKDKIKKFMEKKSYDFPVVLDERQEVAQNYGVQAFPTTLFIDEEGFVYSGIQGPMTDDIMRKELKIK
WP_011948056.1, *C. botulinum* A str. ATCC3502	
Gblock7	TTTCACACAGGAAACAGACC**ATG**AAAAAGATTTGGCTGGCGCTGGCTGGTTTAGTTTTAGCGTTTAGCGCATCGGCGGAAAGTAAGCAAGAGGAGA ACAAACGCGAGAACTCCACGGACAAGGAAGAGAACTCAGACGGCAATGATTCTCAGAAAGACAGTAACAACGATAACAACGACAAAGGATCAAATG ATGAAGATCGCATCAAGAGTATTGATTTCACCCTTACTGATCAATATGGGAAAACTCATAAATTGAGCGACTATGAGGGTAAAGTCGTCTTTCTGAATT TTTGGGCGACCTGGTGCCCGCCGTGCAAGGAGGAAATGCCCTATATTGAACAACTTTATAAAGACTACAACAAGAATAATGATGATGTCGTCATTCTG GGCGTCGCCTCACCCAATTTAGGTCGCGAAGGGTCGCGCGAACACGTTGTAAACTTCTTAAAAGATCAAGGCTACACGTTTCCTGTAGTATTGGATG AAGATGGCGCGTTGGCTTACCAATATGGAATTAACGCTTTCCCTACAACTTTCATCATTGACAAAGAAGGATACGTGACTCAGTACATCCCAGGTGCC ATGGACAAGGCGACAATGGCTTCTTTTATCGAAAACCAGCGCAACAAG**TGA**GAATTCGAGCTCGGTACCCG Protein sequence **MKNNKRYIYISVIMLALLVGVKFGY**DYLSNNYKSNEAINNVSDENSSFQPAVDFTVYDKDNNEVKLSDYKGKKAIVVNFWASWCSPCKYEMPYFQEATNK YNNEDLEILMVNLTDGMRETKGSAEGFMKEEGYDMNVMFDINLDAANKYQLNAIPRTVFIDKEGNLVYDHVGIINKEILDENINKIIN
CbDsbA (WP_011948075.1, *C. botulinum* A str. ATCC3502)	
Gblock8	TTTCACACAGGAAACAGACC**ATG**AAAAAGATTTGGCTGGCGCTGGCTGGTTTAGTTTTAGCGTTTAGCGCATCGGCGTCTCTTAACATTAAGGTCTAC TTCGATTTCGTGTGCCCGTTCTGTTTCTTGGGAGAGGAGTCCCTTAGTGAAGCAATTAAGGGTAAAGACGTGAATATCCAATGGATGCCCTTCGAGCT GCGTCCTGAACCCTCGCCGCGCATTGACCCGTGGAATGACCCTAGTAAGTTAAACGCTTGGAATAACTTCATTGACCCAATCGCGAACAAGCTTGGG ATCGACATGAAGTTACCCAAATTGTCTCCCCACCCGTATACCAATTTAGCCTTTGAGGGATACCATTATGCAAGTGACCATGGAAAGGGTGACGAATATA TCAAGCGTGTTTTCAAGGGTTTTTTTCAAGAAGAGTTAGATATTGGAAAAATCGAAATCCTGGCGAACTTAAGCGAGGAGATCGGTCTGAACAAAGAG GAATTTATTAAGGTATTAAAGAACCGTAAGTACAAAGATAAACAGGAGAAAGCCTTGAAACATGCTTATGAAGAAGCCAACATCACAGCAGTGCCGACA ATGATTATCGGCGACGAAGTTGTTCAAGGAAACACTTCAAAGGAGTCGCTTGAAAAGATCATCAACAAACAGCTTATCAAGAACAAT**TGA**GAATTCGAG CTCGGTACCCG Protein sequence (signal sequence was not removed) **MSLNIKVYFDFV**C**PF**C**FLGEESLSEA**IKGKDVNIQWMPFELRPEPSPRIDPWNDPSKLNAWNNFIDPIANKLGIDMKLPKLSPHPYTNLAFEGYHYASDHGK GDEYIKRVFKGFFQEELDIGKIEILANLSEEIGLNKEEFIKVLKNRKYKDKQEKALKHAYEEANITAVPTMIIGDEVVQGNTSKESLEKIINKQLIKNN
AJP11210.1, *C. difficile* 630	
Gblock9	TTTCACACAGGAAACAGACC**ATG**AAAAAGATTTGGCTGGCGCTGGCTGGTTTAGTTTTAGCGTTTAGCGCATCGGCGAAGGAGAGCAAACCAGTTGA GAAGAATAAAAATAATTCTGAATGGTTCTCGAACTTGGACACCGAGGATGTCGATGGAAACAAAGTAACTAAGGAGATTTTTTCCAACAAGGAGCTGAC CCTGATTAATGTCTGGACAACGTGGTGTGGCCCCTGCGTGGGAGAGATGCCAGAGCTTGAGGCGTTATCTAAAGAGTACGAGTCAAACAACAGCAAC GTTGCAATTAAGGGCTTGGTTGTCGAAGTCGACAAGACAGATATGCGTACTGGATTGTCAAACGAAGAGAAGAATTTGGTAAAAGATATTATGAAAAAAA GTGACGCAACCTATCAGCAGCTGACCGTAAGTGAGGAGCTGAAAAAAACAGACTTCAAGCGTGTGATGGAGTTCCCGACGACTTATTTTGTGGACAAG AAGGGTAACTTCGTCGGTGAAAAGGTTGTGGGTGCCAACTCTAAAGAAGACTGGAAAAAAATCATCGATGAGCGCTTGAAAATGGTTAAGGCAAATGAA **TGA**GAATTCGAGCTCGGTACCCG Protein sequence **MKRFFIVVLG**C**I**C**VLGMISGCSK**KESKPVEKNKNNSEWFSNLDTEDVDGNKVTKEIFSNKELTLINVWTTWCGPCVGEMPELEALSKEYESNNSNVAIKGLVV EVDKTDMRTGLSNEEKNLVKDIMKKSDATYQQLTVSEELKKTDFKRVMEFPTTYFVDKKGNFVGEKVVGANSKEDWKKIIDERLKMVKANE
AJP11595.1, *C. difficile* 630	
Gblock10	TTTCACACAGGAAACAGACC**ATG**AAAAAGATTTGGCTGGCGCTGGCTGGTTTAGTTTTAGCGTTTAGCGCATCGGCGCAAAAAAGTAACGAGAATAATAA GATCAAGGAAGGACAGTCTATCTCTAGCGAGGAAGCAGGCATGTCTATCAACGCGAATGGAAAGAACGAGACATTCAAACCCTCAGATTATACTCTGGAA GCCAAAAAAGAATACGTGTATGAATATCTGGGTCTGAAGTTTAAATTATCCGACAAGTTCCGCAATTATATCGCCGATAAGAAGATCGCGATGCTGGATGAT CAATCTCCTATTGATAAGGAATTGAAGTTGCAATCCTTACATTCGAAAAGATGACAGAAGAGCAAAAGAACGCGGTAATTGAAAAGATGGGCGATGGGTAT AAGAATTGGCAAAATGAGTTGGAGCGTATCGGTACGATTGGCATTTTTGAGAAGAACACCTCAGAGGAGAAAATTTCAAAGATCACAAAATGCGATACACA TACCAAAATTGGGGTCAGCTCAGATGGGAAATATGACTGTTACCTGAGCACCAACTCCGGGGCTGAGTCTAATTTGCTGGACGAGTTGAAGCGTACAGAGA TCCAGATTATTGACAAAAAAGAGCGCCCGAAGAACGGTTTCGTATTGTCTGAGAAGACTGACTTAGAAAACACCGAAGCCTTTAATAAGGAGTCAGTGAAA GACCTTCGTAAGTTAAGCACTAAAGACATCAATGGGAAAGATTTTACCTCCAAAGACTTTGAGAAATACGACCTGACTATGGTGAATGTCTTTGCCACATGGT GCACCGCTTGCGTTAAGGAGATCCCAGACTTAGTTGAGGTGCAGAACGAGATGAAGAGTAAGGGTGTGAACATCGTAGGTGTCGTTACCGATACGGTAGAT GATAATGGTGAGAATAAAGAGGCTATCGAAAAATCCAAGTTGATCCATGAAAAAACAAAAGCCTCTTACCCCTTTTTGATGCCTGATAAGACCAACTTTAACG GGCGCCTGAATGGTATTCAGGCTATGCCGGAGACTTTCTTCGTCGACAGCAACGGTAATATCGTCGGCGACACGTACTCTGGCGCAAAAAGCGCCAAAGAA TGGAAGCAGGTCATTGAGAAAGAGTTAGAGAAAATCAAGAATAAG**TGA**GAATTCGAGCTCGGTACCCG Protein sequence **MKKIVSRGA**C**LLLAL**C**LSLNAVG**CQKSNENNKIKEGQSISSEEAGMSINANGKNETFKPSDYTLEAKKEYVYEYLGLKFKLSDKFRNYIADKKIAMLDDQSPIDKEL KYAILTFEKMTEEQKNAVIEKMGDGYKNWQNELERIGTIGIFEKNTSEEKISKITKCDTHTKIGVSSDGKYDCYLSTNSGAESNLLDELKRTEIQIIDKKERPKNGFVLS EKTDLENTEAFNKESVKDLRKLSTKDINGKDFTSKDFEKYDLTMVNVFATWCTACVKEIPDLVEVQNEMKSKGVNIVGVVTDTVDDNGENKEAIEKSKLIHEKTKASY PFLMPDKTNFNGRLNGIQAMPETFFVDSNGNIVGDTYSGAKSAKEWKQVIEKELEKIKNK
WP_09903396.1, *C. difficile* 630	
Gblock11	TTTCACACAGGAAACAGACC**ATG**AAAAAGATTTGGCTGGCGCTGGCTGGTTTAGTTTTAGCGTTTAGCGCATCGGCGGAAAGTAAACAGGAAGAAAATAAACG CGAAAATAGCACTGATAAAGAGGAGAATTCAGACGGTAACGATTCTCAGAAAGATAGCAACAATGATAATAACGACAAAGGGTCCAATGACGAAGACCGTATTA AATCCATTGACTTCACTCTTACGGATCAATATGGTAAGACTCATAAGCTTTCAGACTATGAGGGTAAGGTGGTATTTCTGAACTTCTGGGCCACCTGGTGCCCCC CCTGTAAGGAGGAAATGCCGTATATCGAGCAGTTGTATAAGGACTACAATAAGAATAATGACGATGTGGTAATTTTGGGGGTTGCGTCACCGAATTTGGGGCGT GAGGGTTCCCGTGAGCACGTTGTGAACTTCTTAAAGGACCAGGGCTACACGTTCCCTGTAGTCTTGGACGAAGACGGTGCTCTTGCCTACCAGTATGGAATCA ATGCCTTCCCGACCACATTCATCATCGATAAAGAGGGCTATGTAACTCAATATATTCCGGGGGCGATGGACAAGGCAACAATGGCTTCTTTTATTGAAAATCAGC GTAACAAA**TGA**GAATTCGAGCTCGGTACCCG Protein sequence (the portion of the protein with 6 transmembrane segments in bold was removed) MQNVN**LFLVFIEGIVSFFSP**C**ILPILPIYLSI**LSNSSVENLKEGKTSFIGSSL**FKNTIFFALGISTTFFILGSSVKVLSMFFNE**NKDL**IMFIGGIIIIIMGLFYMGII**KSSILNREKR FNV**KFKEKAITAFILGFTFSF**GWTPCIGPILASVLVMVSSSSNHLSANLLIAVYTIGFILP**FIITAMFYSKLFKTIDKIKS**NMEIIKKIGGIILIVSGIL**MMVNGFGSISKHFNTSQ** **NSKI**ESKQEENKRENSTDKEENSDGNDSQKDSNNDNNDKGSNDEDRIKSIDFTLTDQYGKTHKLSDYEGKVVFLNFWATWCPPCKEEMPYIEQLYKDYNKNNDDVV ILGVASPNLGREGSREHVVNFLKDQGYTFPVVLDEDGALAYQYGINAFPTTFIIDKEGYVTQYIPGAMDKATMASFIENQRNK
CdDsbA (EQE38533.1, *C. difficile* CD40)	
Gblock16	GAAACAGACC**ATG**AAAAAGATTTGGCTGGCGCTGGCTGGTTTAGTTTTAGCGTTTAGCGCATCGGCGAAGCATTTCAATACCTCACAGAACTCTAAGATCGAGT CGAAACAGGAGGAGAACAAACGCGAGAATAGCACCGATAAAGAAGAGAACAGCGACGGGAACGATAGCCAAAAAGACTCCAACAATGACAACAATGATAAAGG CTCAAACGACGAGGACCGCATTAAGTCTATTGACTTTACTCTGACTGACCAATACGGAAAAACACATAAGTTGTCTGATTATGAGGGCAAGGTGGTCTTCTTAAAT TTTTGGGCCACATGGTGTCCACCTTGCAAGGAGGAAATGCCGTACATCGAACAACTGTATAAAGACTATAACAAGAACAATGACGATGTGGTAATTCTGGGCGTT GCCTCGCCCAACTTAGGCCGCGAAGGGAGCCGTGAACATGTTGTCAACTTTCTTAAAGACCAAGGATATACCTTTCCGGTGGTATTAGACGAAGATGGGGCTTT AGCATATCAGTACGGCATCAACGCCTTTCCCACCACGTTTATCATCGACAAAGAGGGCTACGTAACACAATATATTCCCGGCGCGATGGACAAAGCCACGATGGC TTCCTTCATCGAAAACCAACGCAACAAA**TGA**GAATTCGAGC Protein sequence **MEIIKKIGGIILIVSGILMMVNGFGSIS**KHFNTSQNSKIESKQEENKRENSTDKEENSDGNDSQKDSNNDNNDKGSNDEDRIKSIDFTLTDQYGKTHKLSDYEGKVVFLN FWATWCPPCKEEMPYIEQLYKDYNKNNDDVVILGVASPNLGREGSREHVVNFLKDQGYTFPVVLDEDGALAYQYGINAFPTTFIIDKEGYVTQYIPGAMDKATMASFIE NQRNK
VTR09364.1, *C. difficile* NCTC13750 strain	
Gblock22	GAAACAGACC**ATG**AAAAAGATTTGGCTGGCGCTGGCTGGTTTAGTTTTAGCGTTTAGCGCATCGGCGTCTGCCCCGATCACTGTAGATATTTGGTCAGACATC GCTTGTCCCTGGTGTTACATCGGCAAGCGCAAATTTGAAGCAGGCTTAGCCGAATTCGCTGGACGCGACGATGTGGAAGTGACATACCACTCGTTCGAGCT TGCTCCCGATACGCCTGTGGACTTTGATGGTTCGGAGGTTGATTTCCTTGTACGCCACAAGGGGATGCCAGCCCAACAAGTCGAGGGAATGCTGGAACAAG TTACTGGCATTGCTGCAGAGGTAGGATTAGATTATGACTTCGATAGCTTGCAACATACCAAGACATTAAAAGCACACGAGGCCTTACACTTCGCCAAAGAACGT GGTCGTCAACTTGATCTGGTGGAACGCTTATTTAAAGCGTACTTCGAGGAAGGGCGCCACGTCGGTCGCCCAGATGAACTGGCAGATTTAGCTGCTGATATC GGATTGGATCGTGACGAGGTCATCGCCGCTCTGGATTCAGGAGCATACGCTCCAGCGGTGGCCGCGGATATCGACCAGGCGCGTGCGTACGGTATTAGTGG AGTGCCCTTCTTTGTAATTTCGGGGAAGTATGGAGTAAGTGGTGCTCAAGCACCTGAGATTTTTACGCAGGCCTTAGACAAGGTGCATGCAGAGGCAGGGGA CCGTGTA**TGA**GAATTCGAGC Protein sequence (no predicted signal sequence) MSAPITVDIWSDIACPWCYIGKRKFEAGLAEFAGRDDVEVTYHSFELAPDTPVDFDGSEVDFLVRHKGMPAQQVEGMLEQVTGIAAEVGLDYDFDSLQHTKTLKAH EALHFAKERGRQLDLVERLFKAYFEEGRHVGRPDELADLAADIGLDRDEVIAALDSGAYAPAVAADIDQARAYGISGVPFFVISGKYGVSGAQAPEIFTQALDKVHAE AGDRV

Cysteine residues are underlined. The signal sequence or TM segment(s) in DsbA proteins (shown in bold) were removed and fused to DsbA’s signal sequence with the exception of WP_011948075.1.

**Table 3.** List of primers used in this study

ID	Sequence 5′ to 3′
PR20	gagcggataacaatttcacacagg
PR21	ctgaaaatcttctctcatccgccaa
PR24	ggtctgtttcctgtgtgaaattgttatccgctcacaattc
PR25	gaattcgagctcggtacccggggatcctctagagtc
PR3	tttcacacaggaaacagaccatgaaaaagatttggctgg
PR8	cgggtaccgagctcgaattcttattttttctcggacagatatttc
PR11	tttcacacaggaaacagaccatgaaaaagatttggctggcgctggctggtttagttttagcgtttagcgcatcggcgatcgtgacgtcgcgcgac
PR12	gctcgaattctcaggatgtcgcggtagc
PR90	ggctgttttggcggatgag
PR91	ggtaccgagctcgaattc
PR92	gagaattcgagctcggtaccacacaaccaattattgaagg
PR93	tgctgcccatctagtatttcccctctttc
PR94	gaaatactagatgggcagcagccatcatc
PR95	tctcatccgccaaaacagcctcagatcagcgtcgaccaatag
PR112	agtgcgggctttttttttcgaccaaaggacacaaccaattattgaaggc
PR113	gtcaggtgcgggcttttttctgtgtttccggtaccgagctcgaattc
PR685	cgtctagacgggtaccgagctcgaattc
PR686	atcatatgcaggccgacgactatacc

All *E. coli* strains were grown in NZ or M63 0.2% glucose. The antibiotic concentrations used were kanamycin 40 µg ml^−1^ and carbenicillin 100 µg ml^−1^. Kanamycin and carbenicillin were purchased from Sigma (USA) and GoldBio (USA), respectively. LMW oxidants were purchased from Sigma: glutathione disulphide (Cat. No. G4376), cystine (Cat. No. 30200) and ergothioneine (Cat. No. E7521).

### *In silico* search

Pfam models [42] were used to search for DsbA (PF01323.12), DsbB (PF02600.8) and VKOR (PF07884.6) homologues in clostridial species. blastp searches were independently done using SdbA, clostridial DsbA, *E. coli* DsbB and clostridial VKOR as query sequences against clostridial proteomes (https://blast.ncbi.nlm.nih.gov/Blast.cgi). Signal sequences and TM segments were predicted using TOPCONS [43] and SignalP4 [44]. SignalP4 was used for Gram-positive bacteria and using sensitive settings.

### Motility assay

Swarming assays were done in M63 0.2% glucose and 0.3% agar supplemented with antibiotics and when indicated LMW thiols. All strains were tested for motility complementation over a range of IPTG induction from 0 to 100 µM IPTG. Consecutive assays for strains that resulted in a motility phenotype were done with the following induction conditions: 10 µM IPTG for LL270, LL608 (expressing *Cd*DsbA), LL29 and LL606 (expressing *Ct*DsbA) strains; 25 µM IPTG for LL23 and LL59 (expressing *Cb*DsbA); and 50 µM IPTG for LL27 (expressing *Cd*VKOR) and LL687 (expressing *Mt*DsbA), while no IPTG was used for strains LL26 (expressing *Cs*VKOR-DsbA), LL16 and LL686 (expressing *Ec*DsbA). Motility assays were done with 50 µM IPTG and 1 µM anhydrotetracycline (aTc) to express both *Mt*DsbA and *Mt*VKOR, respectively, in the LL103 strain. To test the Δ*dsbA*Δ*dsbB* mutant, motility plates were supplemented with increasing concentrations of LMW oxidants up to 1 mM. Once the best concentrations were found, plates were supplemented with 500 µM glutathione disulphide, 10 µM cystine or 10 µM ergothioneine. Bacteria were stabbed into plates and incubated at 30 °C. Halos were measured after 48 h.

## Results

We performed an *in silico* search for DsbA, DsbB and VKOR homologues in Clostridia using Pfam models [42,45] as well as blastp [46] using the Gram-positive and facultative anaerobic *S. gordonii* SdbA protein, which has been experimentally demonstrated to be a DsbA catalyst [17]. We identified one DsbA candidate in *C. tetani*, two candidates in *C. botulinum* and five homologues in various *C. difficile* strains (Table 4). These DsbA proteins exhibit conservation in the catalytic region including the CXXC motif and the cis-Proline (c-Pro) residue (Fig. 1e). Additionally, we identified one VKOR protein in the reference strain NCTC13750 of *C. difficile* and a VKOR fused to DsbA in *Clostridium* sp. (Table 4). This protein resembles the VKOR-DsbA protein found in the cyanobacterium *Synechococcus* sp. [12,47] (Fig. 1f). The CXnC motif in the VKOR homologues is also conserved, consistent with VKOR proteins from *M. tuberculosis* and *Synechococcus* sp. (Fig. 1f).

**Table 4.** Clostridial homologues of DsbA and VKOR enzymes

Name	NCBI reference #	UniProt	Organism	Strain	Protein used for search
**VKOR homologues**					
*Cd*VKOR	VTR07230.1		*C. difficile*	NCTC13750	Pfam VKOR
*Cs*VKOR-DsbA	R7I132_9CLOT	R7I132	*Clostridium* sp.	CAG:768	Pfam VKOR
**DsbA homologues**					
–	WP_011948056.1		*C. botulinum A*	ATCC3502	SdbA
*Cb*DsbA	WP_011948075.1	A5HYH8	*C. botulinum A*	ATCC3502	Pfam DsbA
–	AJP11210.1		*C. difficile*	630	SdbA
–	AJP11595.1		*C. difficile*	630	SdbA
–	WP_09903396.1		*C. difficile*	630	WP_011948056.1
*Cd*DsbA	EQE38533.1		*C. difficile*	CD40	AAO34798.1
–	VTR09364.1		*C. difficile*	NCTC13750	WP_011948075.1
*Ct*DsbA	AAO34798.1	Q899M6	*C. tetani*	E88	SdbA

– indicates no protein name assigned based on the findings of this work.

We obtained the codon-optimized genes of the ten *dsb* candidate genes and cloned them under an IPTG-inducible promoter. For the clostridial *dsbA* homologues, we replaced their predicted transmembrane (TM) segments or signal sequences (ss) by *E. coli* DsbA’s signal sequence to ensure proper export in *E. coli* [48]. For WP_011948075.1, the predicted signal sequence was not removed given that it contains the CXXC motif (Table 2, see Discussion).

We tested the ability of these candidates to complement disulphide bond formation in the *E. coli* Δ*dsb* mutants. Mutations of *dsbA* or *dsbB* in *E. coli* are unable to swarm in soft agar plates due to the misfolding of the flagellar P-ring protein, FlgI, which requires a disulphide bond to be functional [9]. Thus, complementation of DsbA’s function can be analysed by looking at motility halos upon induction of the candidate protein.

**Fig. 2.**
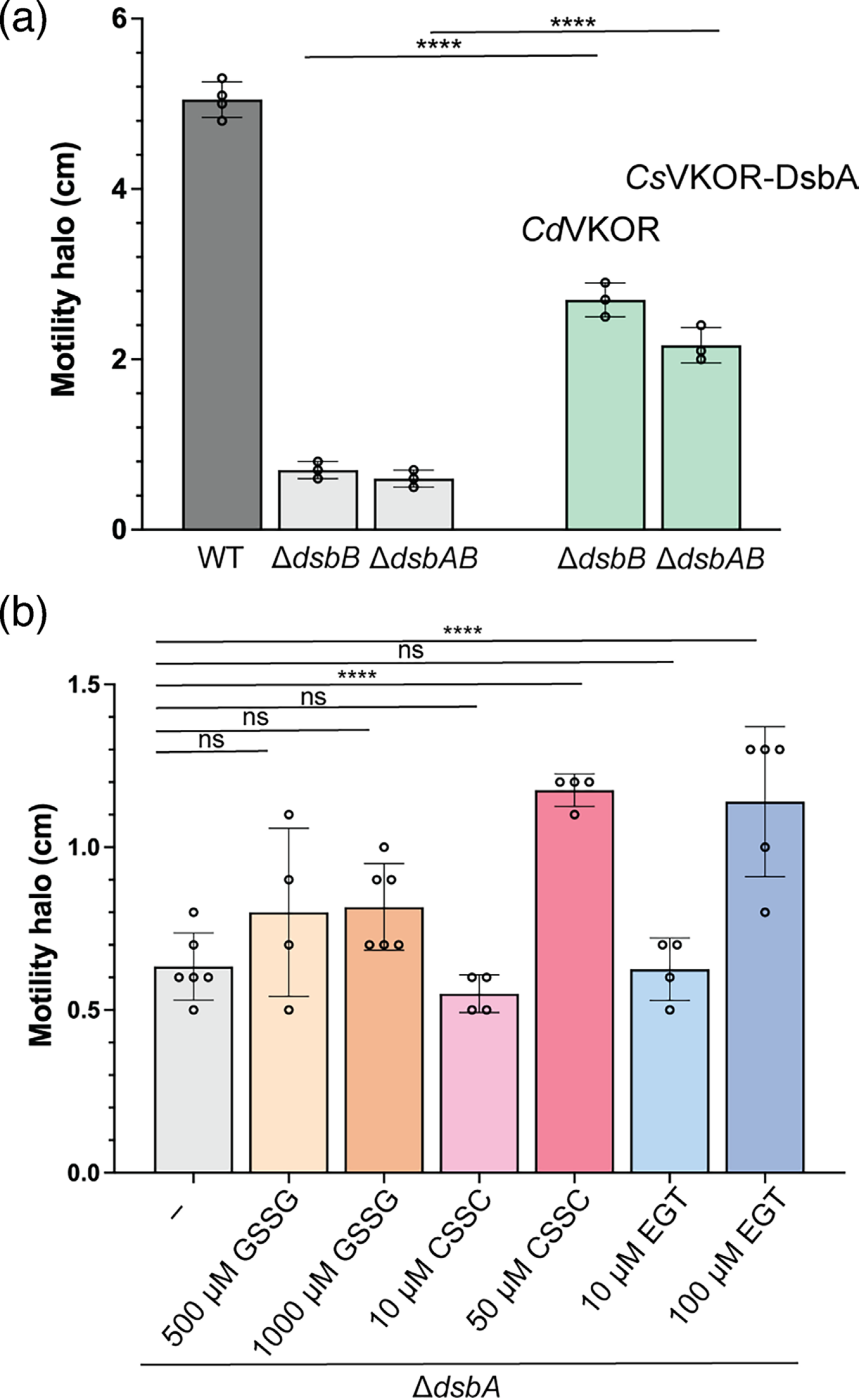
*C. difficile* NCTC13750 VKOR and *Clostridium* sp. VKOR-DsbA fusion complement *E. coli* disulphide bond formation. (**a**) Disulphide bond formation is required for the folding of FlgI, a flagellar P-ring protein, which requires one disulphide bond to be properly folded. The expression *Cd*VKOR and *Cs*VKOR-DsbA in the Δ*dsbB* and Δ*dsbA*Δ*dsbB*, respectively, restores the motility defect of *dsb* mutants. Strains used include CL499, CL379, LL40, LL27 and LL26. Data represents the average ±sd of at least three independent replicas. A statistical test was done using ordinary one-way ANOVA Tukey’s multiple comparisons test. *P*-values are depicted in GP style: ≤0.0001 (****), 0.0002 (***), 0.021 (**), 0.0332 (*) and non-significant (ns). (**b**) High concentrations of LMW oxidants can restore FlgI folding independent of Dsb catalysts to the Δ*dsbA* mutant. LMW thiols include glutathione disulphide (GSSG), cystine (CSSC) and ergothioneine (EGT). Strain used includes LL40. Data represents the average ±sd of at least three independent experiments. A statistical test was done using ordinary one-way ANOVA Dunnett’s multiple comparison test. *P*-values are depicted in GP style: ≤0.0001 (****), 0.0002 (***), 0.021 (**), 0.0332 (*) and non-significant (ns). Swarming halos were measured in M63 glucose media with 0.3% agar after 48-h incubation at 30 °C.

We found that both *C. difficile* VKOR (VTR07230.1, *Cd*VKOR) complemented the Δ*dsbB* and *Clostridium* sp. VKOR-DsbA (R7I132_9CLOT, *Cs*VKOR-DsbA) fusion complemented the double Δ*dsbA*Δ*dsbB* mutant (Fig. 2a). Thus, these VKOR proteins can participate in periplasmic oxidative protein folding.

**Fig. 3.**
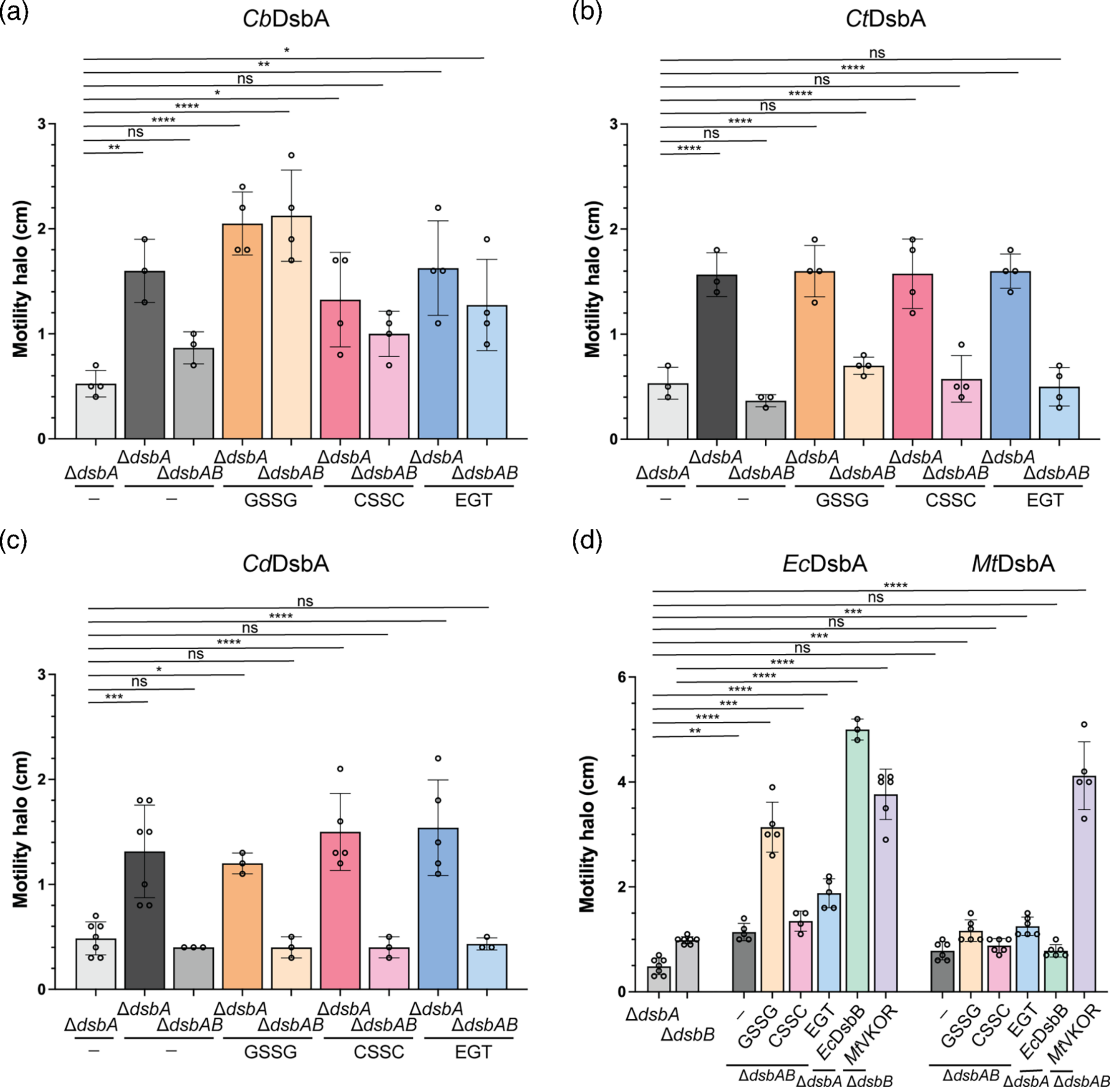
*C. botulinum*, *C. tetani* and *C. difficile* DsbA proteins complement *E. coli* disulphide bond formation. (**a**) Expression *Cb*DsbA in Δ*dsbA* and Δ*dsbA*Δ*dsbB* restores motility. In Δ*dsbA, E. coli* DsbB protein oxidizes *Cb*DsbA to regenerate its activity. In the double Δ*dsbA*Δ*dsbB* mutant, the exogenous addition of glutathione disulphide and ergothioneine regenerates *Cb*DsbA’s function. Strains used include LL40, LL23 and LL59. Concentrations of LMW thiols include 500 µM glutathione disulphide (GSSG), 10 µM cystine (CSSC) and 10 µM ergothioneine (EGT). (**b**) Expression *Ct*DsbA in the Δ*dsbA* mutant restores *E. coli* motility. The addition of LMW oxidants is unable to regenerate *Ct*DsbA’s function. Strains used in this assay include LL40, LL29 and LL606. Concentrations of LMW thiols are the same as (a). (**c**) *Cd*DsbA restores motility in the Δ*dsbA* mutant, while LMW oxidants are unable to regenerate *Cd*DsbA’s function. Strains used in this assay include LL40, LL270 and LL608. Concentrations of LMW thiols are the same as (a). (**d**) *Ec*DsbA can be regenerated by GSSG and EGT when expressed in *E. coli* Δ*dsbA*Δ*dsbB* mutant*,* while *Mt*DsbA is modestly regenerated by GSSG and EGT. Both *Ec*DsbA and *Mt*DsbA are also restored by their respective partners, *Ec*DsbB and *Mt*VKOR. Strains used in this assay include LL40, CL379, LL686, LL16, CL382, LL687, LL103 and LL18. Concentrations of LMW thiols are the same as (a). Data in all graphs represent the average ±sd of at least three independent replicas. Statistical tests were done using ordinary one-way ANOVA Dunnett’s multiple comparisons test. *P*-values are depicted in GP style: ≤0.0001 (****), 0.0002 (***), 0.021 (**), 0.0332 (*) and non-significant (ns). Swarming halos were measured in M63 glucose media with 0.3% agar after 48-h incubation at 30 °C.

Next, we tested the complementation of the clostridial *dsbA* candidates. From *C. botulinum* strain A, only *Cb*DsbA (WP_011948075.1, Fig. S1, available in the online Supplementary Material) complemented the Δ*dsbA* mutant even at low concentrations of IPTG (Figs 3a and S2, bar 2), indicating that this DsbA can be regenerated by *E. coli* DsbB. However, since we were unable to find a homologue of the regenerating partner in *C. botulinum*’s genome, we tested the ability of LMW thiols to regenerate DsbA’s activity. To do this, we expressed *Cb*DsbA in the Δ*dsbA*Δ*dsbB* mutant and added three LMW thiols to the minimal media at concentrations that cannot restore disulphide bond formation to the Δ*dsbA* mutant. It has been previously reported that cystine can restore disulphide bond formation and motility at 100 µM or higher [9]. We found that the addition of 50 µM cystine and 100 µM ergothioneine or higher can restore motility to the Δ*dsbA* mutant, while 1 mM of glutathione disulphide cannot (Fig. 2b). Thus, we tested the regeneration of *Cb*DsbA at low concentrations of cystine (10 µM) and ergothioneine (10 µM) as well as a higher concentration of glutathione disulphide (500 µM). Induction of only the *Cb*DsbA in the Δ*dsbA*Δ*dsbB* mutant failed to fully restore motility (Fig. 3a, bar 3). However, the addition of glutathione disulphide and ergothioneine but not cystine was able to regenerate DsbA’s activity and increase motility of Δ*dsbA*Δ*dsbB* (Fig. 3a, bars 5, 7 and 9). A comparable concentration of glutathione disulphide (10 µM and up to 100 µM) was insufficient to complement motility (Fig. S3). Thus, the higher motility observed under this condition is perhaps the result of the increase of glutathione disulphide concentration in the periplasm (see Discussion). Altogether, these data suggest that *Cb*DsbA can oxidize substrate proteins both in the presence of a regenerating enzyme and in the presence of LMW thiols. Among the tested oxidants and under the tested concentrations, glutathione disulphide most effectively restored *Cb*DsbA activity, while ergothioneine supported only a modest recovery and cystine did not restore activity, indicating variable efficiency of these thiols in DsbA regeneration.

We then tested the DsbA candidate from *C. tetani* E88 and found that *Ct*DsbA (AAO34798.1) can restore motility of the Δ*dsbA* mutant at low concentrations of IPTG (Fig. 3b, bar 2), indicating that *Ct*DsbA can be regenerated by *E. coli* DsbB. However, when we tested its ability to be regenerated by the three LMW thiols in the Δ*dsbA*Δ*dsbB* mutant, we found that it was unable to restore motility (Fig. 3b, bars 5, 7 and 9). Thus, DsbB but not LMW thiols is able to restore *Ct*DsbA’s activity under the tested concentrations.

We next examined the *C. difficile* DsbA-like proteins and found that the homologues from strains 630 and NCTC13750 (AJP11210.1, AJP11595.1, WP_09903396.1 and VTR09364.1) were unable to restore motility to the Δ*dsbA* mutant (Fig. S1). Only *Cd*DsbA (EQE38533.1) from strain CD40 was able to do so (Fig. 3c, bar 2). However, when we expressed *Cd*DsbA in the Δ*dsbA*Δ*dsbB* mutant, it was unable to sustain FlgI oxidation with or without the addition of LMW thiols under the tested concentrations (Fig. 3c, bars 3, 5, 7 and 9). Therefore, both *Cd*DsbA and *Ct*DsbA may either require a different LMW oxidant or an enzyme partner to be regenerated. We were unable to find DsbB or VKOR homologues in the respective predicted proteomes of *C. difficile* CD40 and *C. tetani* E88 strains. To test whether the remaining inactive clostridial DsbA proteins from strains 630 and NCTC13750 could be activated by co-expressing a clostridial VKOR, we cloned *Cdvkor* downstream of each *dsbA* gene and transformed the plasmids into the double Δ*dsbA*Δ*dsbB* mutant. We found that none of the inactive DsbA homologues were able to restore motility when co-expressed with *Cd*VKOR under the tested IPTG concentrations (Fig. S4). Thus, it remains unclear whether these four candidates are functional DsbA proteins or whether they require a specific oxidant.

To compare our results with other well-studied DsbA homologues, we expressed *E. coli* DsbA (*Ec*DsbA) and *M. tuberculosis* DsbA (*Mt*DsbA). Expressing *Ec*DsbA in the Δ*dsbA*Δ*dsbB* mutant can be regenerated by glutathione disulphide (Fig. 3d, bar 4), cystine (Fig. 3d, bar 5) and ergothioneine (Fig. 3d, bar 6), in addition to its native partner, *Ec*DsbB (Fig. 3d, bar 7) and *Mt*VKOR (Fig. 3d, bar 8). In contrast, expressing *Mt*DsbA in *E. coli* Δ*dsbA*Δ*dsbB* mutant*, Mt*DsbA is only regenerated by its native partner *Mt*VKOR (Fig. 3d, bar 14) and moderately by glutathione disulphide (Fig. 3d, bar 10) and ergothioneine (Fig. 3d, bar 10) but not by cystine (Fig. 3d, bar 11) nor *E. coli* DsbB (Fig. 3d, bar 13). Therefore, *C. tetani* and *C. difficile* DsbA proteins may similarly require a dedicated enzyme partner to transfer the electrons from the substrate.

**Fig. 4.**
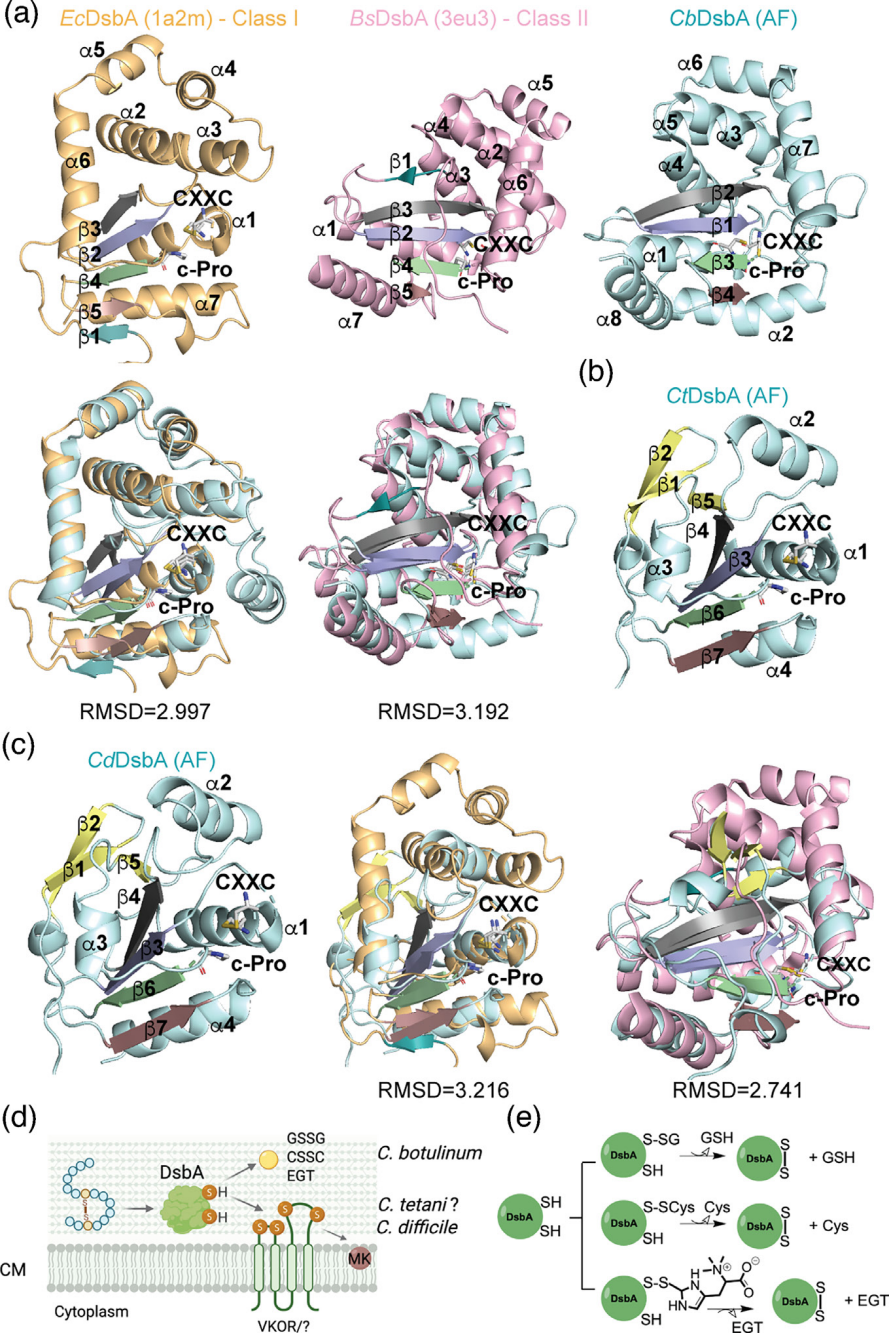
Clostridial disulphide bond-forming enzymes. (**a**) Structural comparison of *E. coli* (1a2m), *B. subtilis* (3eu3) and *C. botulinum* DsbA (AF-A5HYH8, pLDDT average is 95.34) [49]. Secondary structural elements as well as DsbA features are indicated, and *β*-sheets are colour coded to highlight similarity in orientation with respect to class I or II DsbA proteins. Structural superimposition of the *C. botulinum* DsbA AlphaFold model to either *E. coli* or *B. subtilis* DsbA proteins was done using PyMOL Molecular Graphics System, version 1.2r3pre, Schrödinger, LLC. RMSD values are indicated. (**b**) Predicted structure of *C. tetani* DsbA (AF-Q899M6, pLDDT average is 96.76) exhibits the absence of *β*1 strand (teal) and the addition of three *β*-sheets (yellow). (**c**) Predicted structure of the soluble domain of *C. difficile* DsbA exhibits the absence of *β*1 strand (teal) and the addition of three *β*-sheets (yellow). AlphaFold 3 [53] was used to model the structure of *C. difficile* DsbA (pLDDT average of soluble domain is 96.73). Structural superimposition of the *C. difficile* DsbA AlphaFold model to either *E. coli* or *B. subtilis* DsbA proteins was done using PyMOL Molecular Graphics System, version 1.2r3pre, Schrödinger, LLC. RMSD values are indicated. (**d**) Clostridial disulphide bond formation pathway. A DsbA-like protein introduces disulphide bonds into exported proteins. DsbA’s activity could then be regenerated by a second enzyme, VKOR, or a yet unidentified enzyme (?). Unlike *C. tetani* and *C. difficile* DsbA proteins, *C. botulinum* DsbA’s activity can also be recycled by LMW oxidants, including glutathione disulphide (GSSG) or ergothioneine (EGT). QH_2_, reduced quinone; grey arrows indicate electron flow. Created in BioRender. (**e**) Proposed model for regeneration of DsbA by LMW thiols adopted from Xiao *et al*. and Zapun *et al*. [73,74].

Lastly, we performed a structural modelling analysis using the AlphaFold [49] predicted structures of the DsbA proteins (Fig. 4a–c). The structure of DsbA proteins contains five *β*-sheets, and divergence in the topology of strand *β*1 defines two classes of DsbA proteins [50]. *E. coli* DsbA (1a2m) [51] belongs to class I, with a *β*-sheet order of *β*3-2-4-5-1, whereas *Bacillus subtilis* DsbA (BdbD, 3eu3) [52] belongs to class II, with a *β*1-3-2-4-5 topology (Fig. 4a) [50]. The AlphaFold predicted structure of *C. botulinum* DsbA (AF-A5HYH8) [49] suggests a *β*-sheet arrangement of *β*2-1-3-4 (Fig. 4a) and lacks an equivalent of *β*1 strand, similar to *S. aureus* DsbA [19,50]. Additionally, *C. botulinum* DsbA superimposes to *B. subtilis* DsbA [52] with root mean square deviation (RMSD) of 3.192 Å and with *E. coli* DsbA with an RMSD of 2.997 Å (Fig. 4a). A comparable RMSD of 3.139 Å was observed when aligned with the reduced form of *E. coli* DsbA (1a2l). Similar RMSD values (3.1–3.7 Å) have been observed for other Gram-positive DsbA proteins aligned to *E. coli* DsbA [50]. In contrast, the AlphaFold structures of *C. tetani* DsbA (AF-Q899M6, Fig. 4b) [49] and *C. difficile* DsbA predicted with AlphaFold 3 [53] (Fig. 4c) reveal three additional *β*-sheets, with a topology of *β*4-3-6-7 and also lacking *β*1-equivalent strand. Furthermore, *C. tetani* DsbA structure does not superimpose to either *E. coli* or *B. subtilis* DsbA, showing RMSD values of 9.96 and 11.5 Å, respectively (Fig. 4b). In contrast, *C. difficile* DsbA superimposes with *B. subtilis* DsbA with an RMSD of 2.741 Å and with *E. coli* DsbA at 3.216 Å (Fig. 4c). The absence of the *β*1 strand in clostridial DsbA proteins − also observed in *S. aureus* DsbA − may facilitate recycling of these enzymes by both membrane-bound oxidoreductases such as DsbB or VKOR, as well as by smaller oxidants.

## Discussion

We identified disulphide bond-forming enzymes in pathogenic Clostridia species through bioinformatic searches. Some *C. difficile* strains harbour VKOR and DsbA homologues, while others contain only a DsbA. Similarly, *C. botulinum* and *C*. *tetani* possess only DsbA, possibly without a regenerating partner. We showed that *C. botulinum* DsbA can be recycled by glutathione disulphide in *E. coli*. However, *C. tetani* and *C. difficile* DsbA proteins were only regenerated by DsbB in *E. coli*, suggesting that these DsbA proteins may require a specific oxidant or enzyme partner for regeneration (Fig. 4d).

Sulphur-containing molecules are important players in redox reactions in the cell. The most ubiquitous is the tripeptide glutathione (Glu-Cys-Gly) synthesized by eukaryotes and many Gram-negative bacteria [54]. Some organisms that lack glutathione rely on other sulphur compounds, such as coenzyme A, homocysteine, cysteine, mycothiol (Actinomycetes), bacillithiol (Firmicutes), *γ*-Glu-Cys (Halobacteria and lactic acid bacteria), trypanothione (trypanosomes), ergothioneine (fungi and mycobacteria) and coenzymes M and B (methanogenic archaea) [54]. While all three species analysed in this work are predicted to possess the synthesis pathway of cysteine, only *C. difficile* has the complete pathway for glutathione synthesis. Possibly, clostridial DsbA proteins evolved in the presence of a specific thiol that we did not test, which may be key to their regeneration.

The regeneration of *C. botulinum* DsbA by excess exogenous glutathione disulphide suggests that the endogenous levels of this LMW thiol in the *E. coli* periplasm are insufficient to restore *C. botulinum* DsbA activity. While the cytoplasmic concentration of reduced glutathione in *E. coli* has been estimated to reach millimolar levels (up to 10 mM) [55], its concentration in the periplasm remains unknown, and its functional role in this compartment is still unclear. Nevertheless, exogenous glutathione disulphide is thought to cross the outer membrane via porins and to be imported into the cytoplasm by specialized transporters, as it is required for recycling of periplasmic glutaredoxin 3 (GrxC) during DsbB-independent disulphide bond formation [56,59]. We found that higher periplasmic concentrations of glutathione disulphide in the periplasm are required to regenerate *Cb*DsbA than those needed for GrxC, suggesting a lower efficiency or different mechanism of thiol-disulphide exchange for *Cb*DsbA in the *E. coli* periplasm.

Recently, Knoke *et al*. show that periplasmic glutathione disulphide can compensate for the absence of DsbA via an unknown mechanism [60]. Direct interaction between the thiols of the periplasmic proteins and glutathione disulphide could produce at low efficiency disulphide bonds [60]. We indeed observed that high concentrations of glutathione disulphide (1 mM) were not able to oxidize FlgI and restore motility in the absence of DsbA, while medium concentrations of cystine and ergothioneine (>50 µM) restored the motility of the *dsbA* mutant. Thus, we hypothesize that at high concentrations and at low efficiency, LMW thiols can oxidize substrates such as FlgI, while below these concentrations and in the presence of a DsbA-like protein, they bind to the catalytic cysteine (Cys30 in *E. coli* DsbA) and recycle its activity by transferring the electrons to their sulphur atoms, releasing reduced glutathione, cysteine or ergothioneine (Fig. 4e).

While we found a DsbA homologue from *C. difficile* CD40, surprisingly none of the three DsbA-like candidates from *C. difficile* 630, the reference strain, alone or in combination with clostridial VKOR complemented *E. coli dsb* mutants. *C. difficile* genome plasticity and high variation between strains have previously been documented, with estimates of the core genome conservation as low as 16% [61]. Although unlikely, it is possible that *C. difficile* DsbA evolved to fold a particular set of substrates and could not recognize *E. coli* FlgI. Uropathogenic *E. coli* and *Salmonella enterica* harbour a DsbA protein, named DsbL, dedicated to fold a particular virulence factor, arylsulfate sulfotransferase [62]. However, *E. coli* and *S. enterica* DsbL proteins can complement the motility of *dsbA* mutant [62,64]. Finding the protein with DsbA function in *C. difficile* 630 will require further investigation. For instance, to study disulphide bond formation in Clostridia, one could use forward genetic screens in these organisms to find the DsbA-like proteins, the enzyme partner or synthesis pathway of the thiol. Using *β*-galactosidase sensor plasmids that we have previously generated for the expression in other organisms than *E. coli* could facilitate this search [65]. When *β*-galactosidase is translocated across the plasma membrane, it would be inactivated by disulphide bonds. Thus, adapting this sensor in Clostridia would allow a blue–white screen to find mutations that disrupt oxidative protein folding.

The presence of VKOR homologues in *C. difficile* and *Clostridium* sp. was intriguing given the lack of quinone synthesis in these organisms. We think that to oxidize VKOR, Clostridia may use the quinones secreted by other organisms in their niche, suggesting that oxidative protein folding of virulence factors and toxins may be dependent on other commensals in the host or environment. Such growth dependence on menaquinones secreted by neighbour micro-organisms has been previously reported in members of the gut microbiome [66]. Thus, an exciting area of study is how interactions between pathogen and commensal occur in the context of oxidative protein folding in these organisms.

Altogether, our work provides the basis for understanding oxidative protein folding in Clostridia. More studies using reverse and forward genetics in the pathogens would be crucial to determine the export of clostridial DsbA and VKOR candidates, substrate folding in the clostridial cell envelope and, overall, how disulphide bond formation occurs in these anaerobic organisms.

## Supplementary material

10.1099/mic.0.001603 Uncited Supplementary Material 1.

## Abbreviations

IDT: Integrated DNA Technologies
LMW: low molecular weight
RMSD: root mean square deviation

## References

[1] Heras B, Shouldice SR, Totsika M, Scanlon MJ, Schembri MA (2009). DSB proteins and bacterial pathogenicity. Nat Rev Microbiol.

[2] Landeta C, Boyd D, Beckwith J (2018). Disulfide bond formation in prokaryotes. Nat Microbiol.

[3] Furniss RCD, Kaderabkova N, Barker D, Bernal P, Maslova E (2022). Breaking antimicrobial resistance by disrupting extracytoplasmic protein folding. Elife.

[4] Kadeřábková N, Furniss RCD, Maslova E, Eisaiankhongi L, Bernal P (2023). Antibiotic potentiation and inhibition of cross-resistance in pathogens associated with cystic fibrosis. bioRxiv.

[5] Bardwell JCA, McGovern K, Beckwith J (1991). Identification of a protein required for disulfide bond formation in vivo. Cell.

[6] Kadokura H, Beckwith J (2009). Detecting folding intermediates of a protein as it passes through the bacterial translocation channel. Cell.

[7] Bardwell JC, Lee JO, Jander G, Martin N, Belin D (1993). A pathway for disulfide bond formation in vivo. Proc Natl Acad Sci USA.

[8] Missiakas D, Georgopoulos C, Raina S (1993). Identification and characterization of the *Escherichia coli* gene dsbB, whose product is involved in the formation of disulfide bonds in vivo. Proc Natl Acad Sci USA.

[9] Dailey FE, Berg HC (1993). Mutants in disulfide bond formation that disrupt flagellar assembly in *Escherichia coli*. Proc Natl Acad Sci USA.

[10] Bader MW, Xie T, Yu CA, Bardwell JCA (2000). Disulfide bonds are generated by quinone reduction. J Biol Chem.

[11] Bader M, Muse W, Ballou DP, Gassner C, Bardwell JCA (1999). Oxidative protein folding is driven by the electron transport system. Cell.

[12] Dutton RJ, Boyd D, Berkmen M, Beckwith J (2008). Bacterial species exhibit diversity in their mechanisms and capacity for protein disulfide bond formation. Proc Natl Acad Sci USA.

[13] Li W (2023). Distinct enzymatic strategies for *de novo* generation of disulfide bonds in membranes. Crit Rev Biochem Mol Biol.

[14] Mejia-Santana A, Collins R, Doud EH, Landeta C (2025). Disulfide bonds are required for cell division, cell envelope biogenesis and antibiotic resistance proteins in mycobacteria. bioRxiv.

[15] Daniels R, Mellroth P, Bernsel A, Neiers F, Normark S (2010). Disulfide bond formation and cysteine exclusion in gram-positive bacteria. J Biol Chem.

[16] Davey L, Halperin SA, Lee SF (2016). Thiol-disulfide exchange in gram-positive firmicutes. Trends Microbiol.

[17] Davey L, Ng CKW, Halperin SA, Lee SF (2013). Functional analysis of paralogous thiol-disulfide oxidoreductases in *Streptococcus gordonii*. J Biol Chem.

[18] Davey L, Cohen A, LeBlanc J, Halperin SA, Lee SF (2016). The disulfide oxidoreductase SdbA is active in *Streptococcus gordonii* using a single C-terminal cysteine of the CXXC motif. Mol Microbiol.

[19] Heras B, Kurz M, Jarrott R, Shouldice SR, Frei P (2008). *Staphylococcus aureus* DsbA does not have a destabilizing disulfide. A new paradigm for bacterial oxidative folding. J Biol Chem.

[20] Neumann-Schaal M, Hofmann JD, Will SE, Schomburg D (2015). Time-resolved amino acid uptake of *Clostridium* difficile 630Δerm and concomitant fermentation product and toxin formation. BMC Microbiol.

[21] Karlsson S, Lindberg A, Norin E, Burman LG, Åkerlund T (2000). Toxins, butyric acid, and other short-chain fatty acids are coordinately expressed and down-regulated by cysteine in *Clostridium difficile*. Infect Immun.

[22] Dubois T, Dancer-Thibonnier M, Monot M, Hamiot A, Bouillaut L (2016). Control of *Clostridium* difficile physiopathology in response to cysteine availability. Infect Immun.

[23] Edwards AN, Karim ST, Pascual RA, Jowhar LM, Anderson SE (2016). Chemical and stress resistances of *Clostridium difficile* Spores and Vegetative Cells. Front Microbiol.

[24] Sievers S, Dittmann S, Jordt T, Otto A, Hochgräfe F (2018). Comprehensive redox profiling of the thiol proteome of *Clostridium difficile*. Mol Cell Proteomics.

[25] Fischer A, Montal M (2007). Crucial role of the disulfide bridge between botulinum neurotoxin light and heavy chains in protease translocation across membranes. J Biol Chem.

[26] Zuverink M, Chen C, Przedpelski A, Blum FC, Barbieri JT (2015). A heterologous reporter defines the role of the tetanus toxin interchain disulfide in light-chain translocation. Infect Immun.

[27] Songer JG, Harmon AE, Keel MK (2016). Toxins of clostridium difficile. Clostridial Diseases in Animals.

[28] Fimlaid KA, Bond JP, Schutz KC, Putnam EE, Leung JM (2013). Global analysis of the sporulation pathway of *Clostridium difficile*. PLoS Genet.

[29] Lawley TD, Croucher NJ, Yu L, Clare S, Sebaihia M (2009). Proteomic and genomic characterization of highly infectious *Clostridium difficile* 630 spores. J Bacteriol.

[30] Kracke F, Vassilev I, Krömer JO (2015). Microbial electron transport and energy conservation - the foundation for optimizing bioelectrochemical systems. *Front Microbiol*.

[31] Dumitrescu DG, Gordon EM, Kovalyova Y, Seminara AB, Duncan-Lowey B (2022). A microbial transporter of the dietary antioxidant ergothioneine. Cell.

[32] Zhang Y, Gonzalez-Gutierrez G, Legg KA, Walsh BJC, Pis Diez CM (2022). Discovery and structure of a widespread bacterial ABC transporter specific for ergothioneine. Nat Commun.

[33] Landeta C, Blazyk JL, Hatahet F, Meehan BM, Eser M (2015). Compounds targeting disulfide bond forming enzyme DsbB of gram-negative bacteria. Nat Chem Biol.

[34] Hatahet F, Blazyk JL, Martineau E, Mandela E, Zhao Y (2015). Altered *Escherichia coli* membrane protein assembly machinery allows proper membrane assembly of eukaryotic protein vitamin K epoxide reductase. Proc Natl Acad Sci USA.

[35] Hibender S, Landeta C, Berkmen M, Beckwith J, Boyd D (2017). Aeropyrum pernix membrane topology of protein VKOR promotes protein disulfide bond formation in two subcellular compartments. *Microbiology (Reading*).

[36] Chavez D, Amarquaye GN, Mejia-Santana A, Ryan K (2024). Warfarin analogs target disulfide bond-forming enzymes and suggest a residue important for quinone and coumarin binding. J Biol Chem.

[37] Ishihara T, Tomita H, Hasegawa Y, Tsukagoshi N, Yamagata H (1995). Cloning and characterization of the gene for a protein thiol-disulfide oxidoreductase in *Bacillus brevis*. J Bacteriol.

[38] Ng TCN, Kwik JF, Maier RJ (1997). Cloning and expression of the gene for a protein disulfide oxidoreductase from Azotobacter vinelandii: complementation of an Escherichia coli dsbA mutant strain. Gene.

[39] Dumoulin A, Grauschopf U, Bischoff M, Thöny-Meyer L, Berger-Bächi B (2005). *Staphylococcus aureus* DsbA is a membrane-bound lipoprotein with thiol-disulfide oxidoreductase activity. Arch Microbiol.

[40] Penning S, Hong Y, Cunliffe T, Hor L, Totsika M (2024). Unveiling the versatility of the thioredoxin framework: insights from the structural examination of *Francisella tularensis* DsbA1. Comput Struct Biotechnol J.

[41] Meyer AJ, Segall-Shapiro TH, Glassey E, Zhang J, Voigt CA (2019). *Escherichia coli* “Marionette” strains with 12 highly optimized small-molecule sensors. Nat Chem Biol.

[42] Mistry J, Chuguransky S, Williams L, Qureshi M, Salazar GA (2021). Pfam: The protein families database in 2021. Nucleic Acids Res.

[43] Bernsel A, Viklund H, Hennerdal A, Elofsson A (2009). TOPCONS: consensus prediction of membrane protein topology. Nucleic Acids Res.

[44] Almagro Armenteros JJ, Tsirigos KD, Sønderby CK, Petersen TN, Winther O (2019). SignalP 5.0 improves signal peptide predictions using deep neural networks. Nat Biotechnol.

[45] Sonnhammer ELL, Eddy SR, Durbin R (1997). Pfam: a comprehensive database of protein domain families based on seed alignments. *Proteins*.

[46] Altschul SF, Madden TL, Schäffer AA, Zhang J, Zhang Z (1997). Gapped BLAST and PSI-BLAST: a new generation of protein database search programs. Nucleic Acids Res.

[47] Li W, Schulman S, Dutton RJ, Boyd D, Beckwith J (2010). Structure of a bacterial homologue of vitamin K epoxide reductase. Nature.

[48] Schierle CF, Berkmen M, Huber D, Kumamoto C, Boyd D (2003). The DsbA signal sequence directs efficient, cotranslational export of passenger proteins to the *Escherichia coli* periplasm via the signal recognition particle pathway. J Bacteriol.

[49] Jumper J, Evans R, Pritzel A, Green T, Figurnov M (2021). Highly accurate protein structure prediction with AlphaFold. Nature.

[50] McMahon RM, Premkumar L, Martin JL (2014). Four structural subclasses of the antivirulence drug target disulfide oxidoreductase DsbA provide a platform for design of subclass-specific inhibitors. BBA - Proteins and Proteomics.

[51] Guddat LW, Bardwell JCA, Zander T, Martin JL (1997). The uncharged surface features surrounding the active site of *Escherichia coli* DsbA are conserved and are implicated in peptide binding. Protein Sci.

[52] Crow A, Lewin A, Hecht O, Carlsson Möller M, Moore GR (2009). Crystal structure and biophysical properties of *Bacillus subtilis* BdbD. An oxidizing thiol:disulfide oxidoreductase containing a novel metal site. J Biol Chem.

[53] Abramson J, Adler J, Dunger J, Evans R, Green T (2024). Accurate structure prediction of biomolecular interactions with AlphaFold 3. Nature.

[54] Poole LB (2015). The basics of thiols and cysteines in redox biology and chemistry. Free Radic Biol Med.

[55] Fahey RC, Brown WC, Adams WB, Worsham MB (1978). Occurrence of glutathione in bacteria. J Bacteriol.

[56] Suzuki H, Koyanagi T, Izuka S, Onishi A, Kumagai H (2005). The yliA, -B, -C, and -D genes of *Escherichia coli* K-12 encode a novel glutathione importer with an ATP-binding cassette. J Bacteriol.

[57] Knoke LR, Muskietorz M, Kühn L, Leichert LI (2025). The ABC transporter Opp imports reduced glutathione, while Gsi imports glutathione disulfide in *Escherichia coli*. *Redox Biol*.

[58] Eser M, Masip L, Kadokura H, Georgiou G, Beckwith J (2009). Disulfide bond formation by exported glutaredoxin indicates glutathione’s presence in the *E. coli* periplasm. Proc Natl Acad Sci USA.

[59] Pittman MS, Robinson HC, Poole RK (2005). A bacterial glutathione transporter (*Escherichia coli* CydDC) exports reductant to the periplasm. J Biol Chem.

[60] Knoke LR, Zimmermann J, Lupilov N, Schneider JF, Celebi B (2023). The role of glutathione in periplasmic redox homeostasis and oxidative protein folding in Escherichia coli. Redox Biol.

[61] Knight DR, Elliott B, Chang BJ, Perkins TT, Riley TV (2015). Diversity and evolution in the genome of *Clostridium difficile*. Clin Microbiol Rev.

[62] Grimshaw JPA, Stirnimann CU, Brozzo MS, Malojcic G, Grütter MG (2008). DsbL and DsbI form a specific dithiol oxidase system for periplasmic arylsulfate sulfotransferase in uropathogenic *Escherichia coli*. J Mol Biol.

[63] Totsika M, Heras B, Wurpel DJ, Schembri MA (2009). Characterization of two homologous disulfide bond systems involved in virulence factor biogenesis in uropathogenic *Escherichia coli* CFT073. J Bacteriol.

[64] Lin D, Kim B, Slauch JM (2009). DsbL and DsbI contribute to periplasmic disulfide bond formation in *Salmonella* enterica serovar *Typhimurium*. *Microbiology*.

[65] Dyotima S, Mendoza J, Landeta C (2024). Development of a sensor for disulfide bond formation in diverse bacteria. J Bacteriol.

[66] Fenn K, Strandwitz P, Stewart EJ, Dimise E, Rubin S (2017). Quinones are growth factors for the human gut microbiota. Microbiome.

[67] Sievers F, Wilm A, Dineen D, Gibson TJ, Karplus K (2011). Fast, scalable generation of high-quality protein multiple sequence alignments using Clustal Omega. *Mol Syst Biol*.

[68] Waterhouse AM, Procter JB, Martin DMA, Clamp M, Barton GJ (2009). Jalview Version 2--a multiple sequence alignment editor and analysis workbench. Bioinformatics.

[69] Kadokura H, Beckwith J (2002). Four cysteines of the membrane protein DsbB act in concert to oxidize its substrate DsbA. EMBO J.

[70] Amann E, Ochs B, Abel KJ (1988). Tightly regulated tac promoter vectors useful for the expression of unfused and fused proteins in Escherichia coli. Gene.

[71] Weiss DS, Chen JC, Ghigo JM, Boyd D, Beckwith J (1999). Localization of FtsI (PBP3) to the septal ring requires its membrane anchor, the Z ring, FtsA, FtsQ, and FtsL. J Bacteriol.

[72] Dutton RJ, Wayman A, Wei J-R, Rubin EJ, Beckwith J (2010). Inhibition of bacterial disulfide bond formation by the anticoagulant warfarin. Proc Natl Acad Sci U S A.

[73] Xiao R, Lundström-Ljung J, Holmgren A, Gilbert HF (2005). Catalysis of thiol/disulfide exchange. Glutaredoxin 1 and protein-disulfide isomerase use different mechanisms to enhance oxidase and reductase activities. J Biol Chem.

[74] Zapun A, Bardwell JC, Creighton TE (1993). The reactive and destabilizing disulfide bond of DsbA, a protein required for protein disulfide bond formation in vivo. Biochemistry.

