# Supplementary material for: Disulphide bond-forming enzymes in clostridial species
Source: Microbiology (Reading). 2025 Sep 12;171(9):001603. doi: 10.1099/mic.0.001603 (PMC12453122; doi:10.1099/mic.0.001603)

Supplemental Information

Disulfide bond-forming enzymes in Clostridial species

Running title: DsbA and VKOR homologs in clostridial species

Claudia Antonika[1], Jocelyne Mendoza[1], and Cristina Landeta[1]*

Author's affiliations:

[1] Department of Biology. Indiana University. Bloomington, USA.

*Correspondence should be addressed to Dr. Cristina Landeta: clandeta@iu.edu

Keywords: disulfide bond, DsbA, DsbB, VKOR, Clostridium botulinum, Clostridium tetani, Clostridioides difficile, low molecular weight thiol, glutathione, cystine, ergothioneine

Supplementary Figure 1. *C. botulinum and C. difficile* DsbA homologs that do not complement disulfide bond formation. Disulfide bond formation is required for the folding of FlgI, a flagellar P-ring protein, which requires one disulfide bond to be properly folded. Strains used include: LL40, LL267, LL268, LL271, LL269 and LL270. Data represents the average ±SD of at least three independent replicas. Swarming halos were measured in M63 glucose media with 0.3% agar after 48 h incubation at 30ºC.

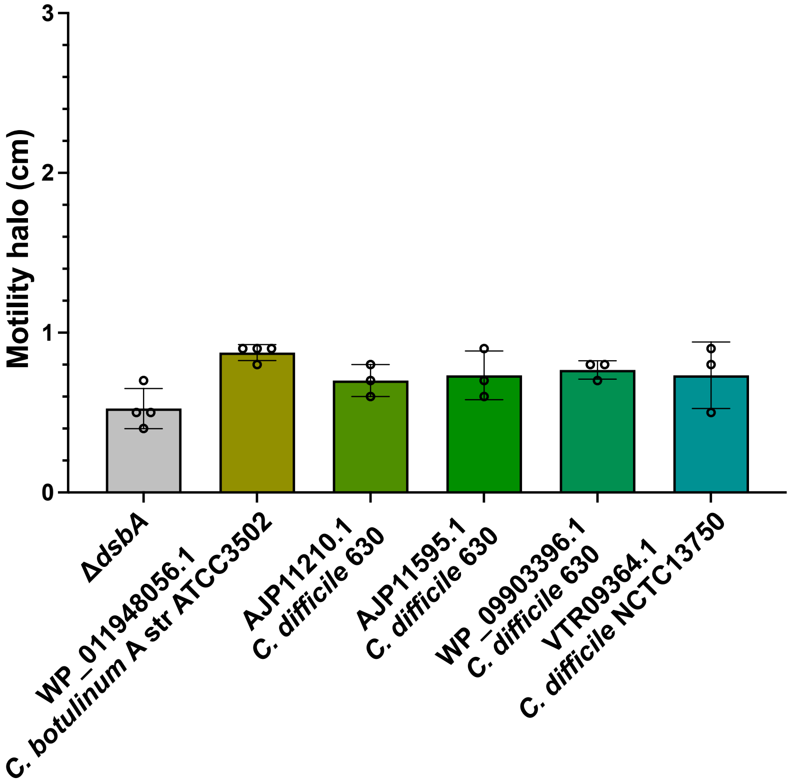

Supplementary Figure 2. *C. botulinum* DsbA homolog complements motility at low inducer concentration (IPTG). Strains used include: LL40 and LL23 under different concentration of IPTG. Data represents the average ±SD of at least three independent replicas. Swarming halos were measured in M63 glucose media with 0.3% agar after 48 h incubation at 30ºC.

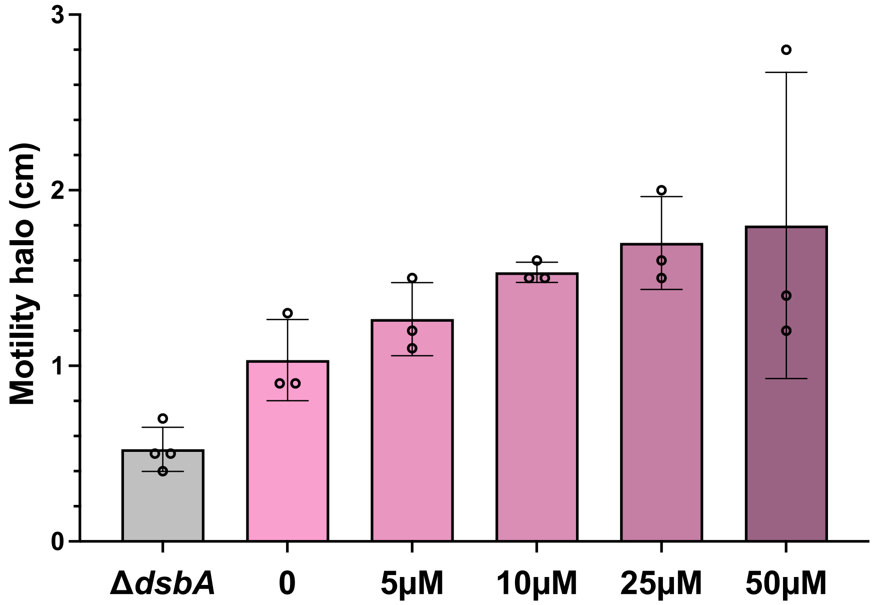

Supplementary Figure 3. Low concentrations of glutathione disulfide (GSSG) are not sufficient to oxidize *C. botulinum* DsbA. Strains used include: LL40 and LL59 under different concentrations of GSSG. Data represents the average ±SD of at least three independent replicas. Swarming halos were measured in M63 glucose media with 0.3% agar after 48 h incubation at 30ºC.

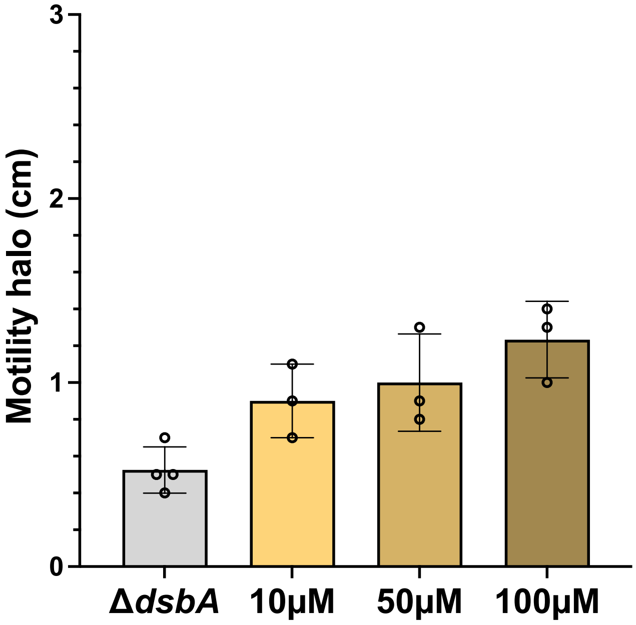

Supplementary Figure 4. Motility complementation of inactive DsbA candidates when co-expressed with *C. difficile* VKOR (VTR07230.1). Δ*dsbA*Δ*dsbB* mutant (HK329 strain) was transformed with plasmids PL429, PL430, PL433, PL431 and PL432, respectively and tested in a range of IPTG concentrations. Data represents the average ±SD of four independent replicas. Swarming halos were measured in M63 glucose media with 0.3% agar after 48 h incubation at 30ºC.

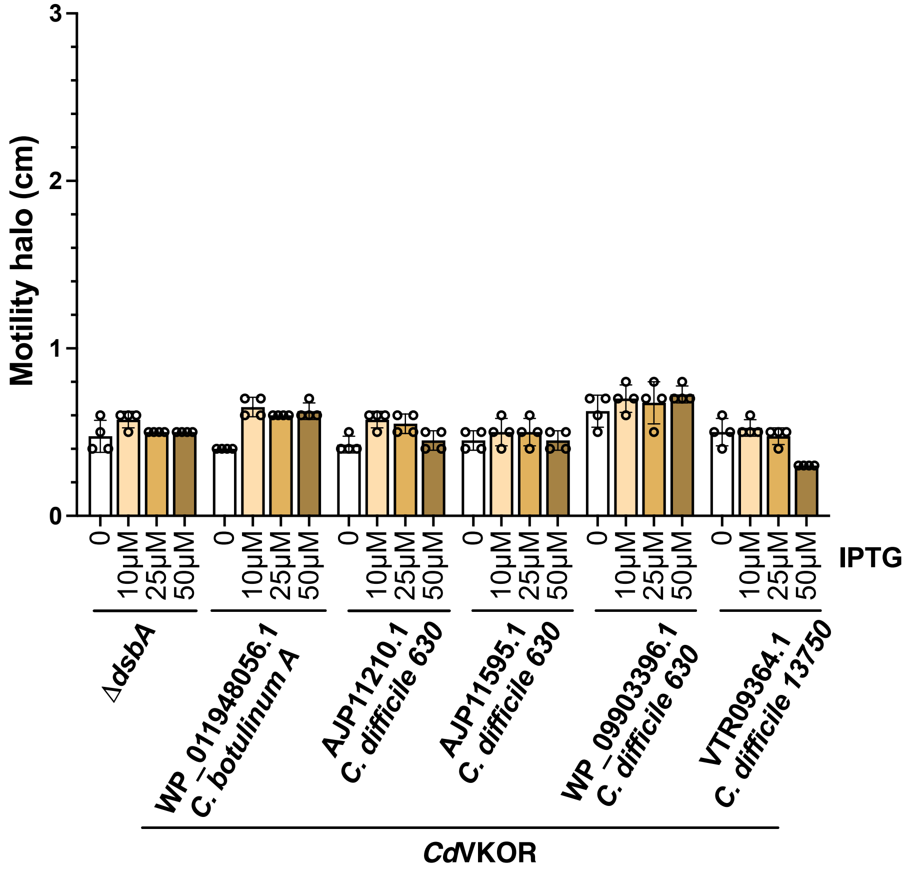

Supplement: Uncited Supplementary Material 1. [file mic-171-01603-s001.pdf]
